# Phylogenetic background and habitat drive the genetic diversification of *Escherichia coli*

**DOI:** 10.1371/journal.pgen.1008866

**Published:** 2020-06-12

**Authors:** Marie Touchon, Amandine Perrin, Jorge André Moura de Sousa, Belinda Vangchhia, Samantha Burn, Claire L. O’Brien, Erick Denamur, David Gordon, Eduardo PC Rocha

**Affiliations:** 1 Microbial Evolutionary Genomics, Institut Pasteur, CNRS, UMR3525, 25-28 rue Dr Roux, Paris, 75015, France; 2 Sorbonne Université, Collège doctoral, F-75005 Paris, France; 3 Ecology and Evolution, Research School of Biology, The Australian National University, Acton, ACT, Australia; 4 Department of Veterinary Microbiology, College of Veterinary Sciences & Animal Husbandry, Central Agricultural University, Selesih, Aizawl, Mizoram, India; 5 School of Medicine, University of Wollongong, Northfields Ave Wollongong, Australia; 6 Université de Paris, IAME, UMR 1137, INSERM, Paris, 75018, France; 7 AP-HP, Laboratoire de Génétique Moléculaire, Hôpital Bichat, 75018, Paris, France; University of Warwick, UNITED KINGDOM

## Abstract

*Escherichia coli* is mostly a commensal of birds and mammals, including humans, where it can act as an opportunistic pathogen. It is also found in water and sediments. We investigated the phylogeny, genetic diversification, and habitat-association of 1,294 isolates representative of the phylogenetic diversity of more than 5,000 isolates from the Australian continent. Since many previous studies focused on clinical isolates, we investigated mostly other isolates originating from humans, poultry, wild animals and water. These strains represent the species genetic diversity and reveal widespread associations between phylogroups and isolation sources. The analysis of strains from the same sequence types revealed very rapid change of gene repertoires in the very early stages of divergence, driven by the acquisition of many different types of mobile genetic elements. These elements also lead to rapid variations in genome size, even if few of their genes rise to high frequency in the species. Variations in genome size are associated with phylogroup and isolation sources, but the latter determine the number of MGEs, a marker of recent transfer, suggesting that gene flow reinforces the association of certain genetic backgrounds with specific habitats. After a while, the divergence of gene repertoires becomes linear with phylogenetic distance, presumably reflecting the continuous turnover of mobile element and the occasional acquisition of adaptive genes. Surprisingly, the phylogroups with smallest genomes have the highest rates of gene repertoire diversification and fewer but more diverse mobile genetic elements. This suggests that smaller genomes are associated with higher, not lower, turnover of genetic information. Many of these genomes are from freshwater isolates and have peculiar traits, including a specific capsule, suggesting adaptation to this environment. Altogether, these data contribute to explain why epidemiological clones tend to emerge from specific phylogenetic groups in the presence of pervasive horizontal gene transfer across the species.

## Author summary

Previous large scale studies on the evolution of *E*. *coli* focused on clinical isolates emphasizing virulence and antibiotic resistance in medically important lineages. Yet, most *E*. *coli* strains are either human commensals or not associated with humans at all. Here, we analyzed a large collection of non-clinical isolates of the species to assess the mechanisms of gene repertoire diversification in the light of isolation sources and phylogeny. We show that gene repertoires evolve so rapidly by the high turnover of mobile genetic elements that epidemiologically indistinguishable strains can be phenotypically extremely heterogeneous, illustrating the velocity of bacterial adaptation and the importance of accounting for the information on the whole genome at the epidemiological scale. Phylogeny and habitat shape the genetic diversification of *E*. *coli* to similar extents. Surprisingly, freshwater strains seem specifically adapted to this environment, breaking the paradigm that *E*. *coli* environmental isolates are systematically fecal contaminations. As a consequence, the evolution of this species is also shaped by environmental habitats, and it may diversify by acquiring genes and mobile elements from environmental bacteria (and not just from gut bacteria). This may facilitate the acquisition of virulence factors and antibiotic resistance in the strains that become pathogenic.

## Introduction

The integration of epidemiology and genomics has greatly contributed to our understanding of the population genetics of epidemic clones of pathogenic bacteria. However, the forces driving the emergence of these lineages in species where most clades are dominated by commensal or environmental strains remain unclear. *Escherichia coli* is a commensal of the gut microbiota of mammals and birds (primary habitat) [1–3], and has been found in host-independent secondary habitats including soil, sediments, and water [4–7]. Yet, some *E*. *coli* strains produce virulence factors endowing them with the ability to cause a broad range of intestinal or extra-intestinal diseases (pathotypes) in humans and domestic animals [8–13]. Many of these are becoming resistant to multiple antibiotics at a worrisome pace [14, 15].

Studies on *E*. *coli* were seminal in the development of bacterial population genetics [16]. They showed moderate levels of recombination in the species [3, 17–19], and a strong phylogenetic structure with eight main phylogroups, among which four (A, B1, B2 and D) represent the majority of the strains and four others (C, E, F and G) are rarer [20–22]. Strains differ in their phenotypic and genotypic characteristics within and across phylogroups [2, 3, 23, 24], and their isolation frequency depends on factors such as host species, diet, sex, age [25–27], body mass [28], but also climate [29, 30], and geographic location [31]. Strains of phylogroups A and B1 appear to be more generalists since they can be isolated from all vertebrates [2] and are often isolated from secondary habitats [7, 32–35]. *E*. *coli* strains able to survive and persist in water environments usually belong to the B1 phylogroup [7, 33, 34]. In contrast, the extraintestinal pathogenic strains usually belong to phylogroups B2 and D [36–38]. Genome size also differs among phylogroups, with A and B1 strains having smaller genomes than B2 or D strains [23].

The phylogenetic vicinity of geographically remote *E*. *coli* isolates, and the co-isolation of phylogenetically distant strains, supports the hypothesis that strains circulate rapidly across the globe [39, 40]. The genome of the species is also remarkably plastic, since only about half of the average genome is present across most strains of the species and the pan-genome vastly exceeds the size of the typical genome [41–44]. Interestingly, the rapid circulation of strains and the high plasticity of their genomes have not erased the associations of certain clades with certain isolation sources. These associations might reflect local adaptation to the isolation habitat [16, 45], which would suggest frequent genetic interactions between the novel adaptive changes and the strains’ genomic background.

Understanding how the evolution of gene repertoires is shaped by population structure and habitats requires large-scale comparative genomics of samples with diverse sources of isolation representative of natural populations of *E*. *coli*. Most of the efforts of genome sequencing have been devoted to study pathogenic lineages and very few genomic data are available for commensal strains, especially in wild animals, and environmental strains. Here, we analysed the genomes of a large collection of *E*. *coli* strains collected across many human, domestic and wild animal and environmental sources in different geographic locations from the Australian continent. This collection is dominated by non-clinical isolates, corresponding to the main habitats of the species. We sought to understand the dynamics of the evolution of gene repertoires and how it was driven by mobile genetic elements. The analysis of the isolation sources in the light of phylogenetic structure and genome variation suggests that rates and mechanisms of adaptation vary with the habitat and the phylogenomic background. This contributes to explain why known epidemiological clones of the species emerge from specific phylogenetic groups, even though virulence strongly depends on the acquisition of virulence factors by horizontal gene transfer.

## Results

### The large and little known pan-genome of *E*. *coli*

**Fig 1:**
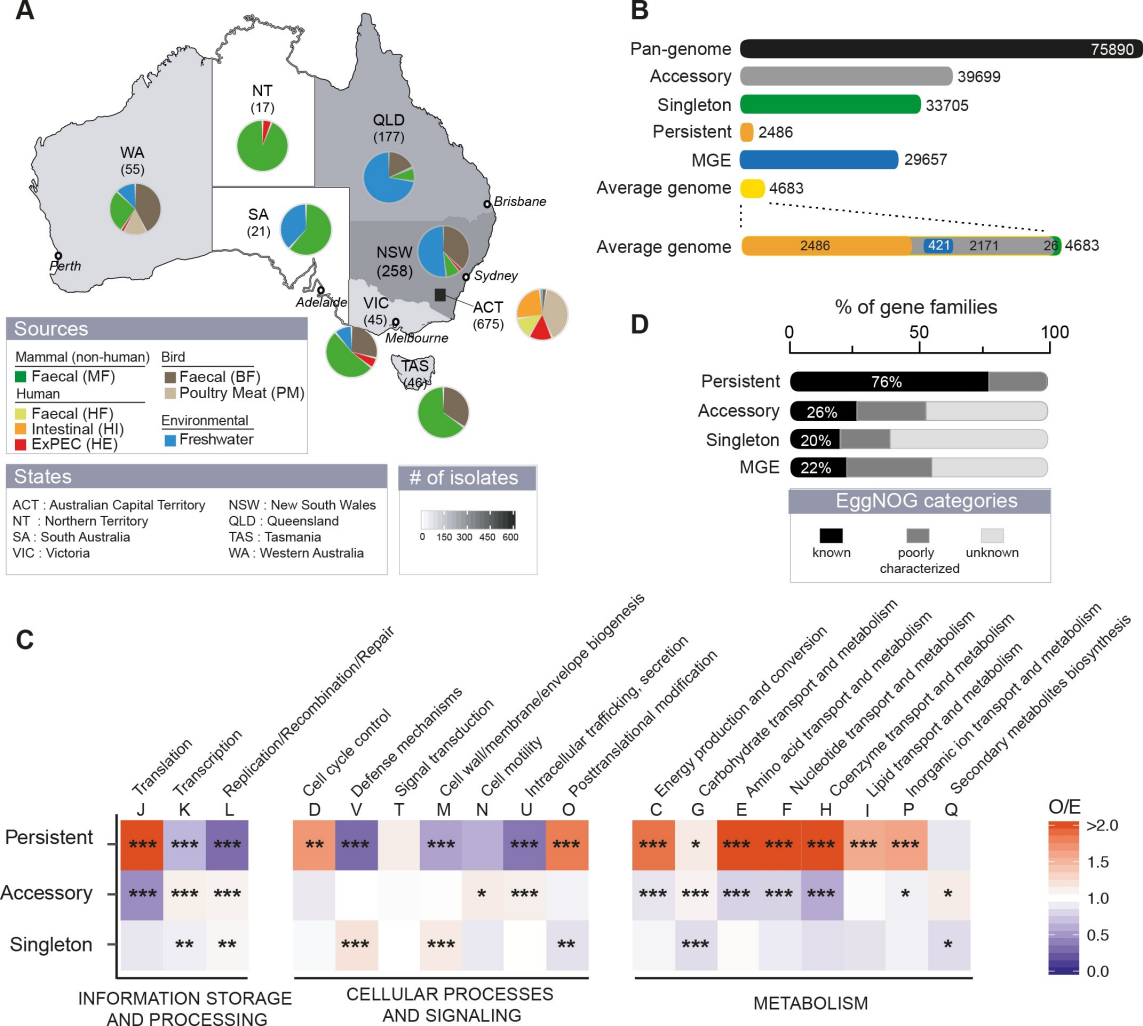
The genetic diversity of Australian *E*. *coli*. **A.** Distribution of isolates per region and per source. **B.** The pan-genome is composed of 75,890 gene families, of which 33,705 are singletons (in green, present in a single genome), 2,486 persistent (in gold, present in at least 99% of genomes), the remaining being accessory (in grey). 29,657 gene families (39% of the pan-genome) were related to mobile genetic elements (MGE). **C.** Functional EggNOG categories of pan-genome gene families. The ratio observed/expected (O/E) for the frequency of non-supervised orthologous groups (NOGs, shown as capitalized letters) is reported for all comparisons with a color code ranging from blue (under-representation) to red (over-representation). The level of significance of each Fisher’s exact test was indicated (P> = 0.05 : ns; P<0.05 : *; P<0.01 : **; P<0.001 :***). It was performed on each 2*2 contingency table. Gene families lacking matches to the EggNOG functional categories were discarded. **D.** Percentage of the different EggNOG categories (see insert) in the persistent, accessory and singleton gene families and among genes associated to MGE.

We sequenced and annotated the genomes of 1,294 *E*. *coli sensu stricto* strains selected from more than 3,300 non-human vertebrate hosts, 1,000 humans and 800 environmental samples between 1993 and 2015, chosen to represent the phylogenetic diversity of the species (Materials and Methods, Fig 1A, S1 Text). All samples were collected by a single team, spanning a 20 year-period, from different regions in a single isolated continent (Australia). The origin of each strain was accurately characterized and the genomes were uniformly annotated and analyzed using the same bioinformatics processes. The strains were isolated from humans, domesticated and wild animals, representing the primary habitat of *E*. *coli*, and from freshwater, representing its secondary habitat [3]. Less than 22% of the samples were recovered from clinical situations. A series of controls confirmed that the sequences were of high quality and contained the known essential genes (S2 Text). The genomes varied widely in size from 4.2 to 6.0 Mb (average 5 Mb), but had similar densities of protein-coding sequences (~87%) and GC content (50.6%, S1 Fig and S1 Table).

The pan-genome contains 75,890 gene families, which is over 16 times the average genome size. The core genome is very small (295 genes), a feature typical of comparisons involving many genomes. As a result, we have opted to focus, whenever possible, on the persistent genome. This corresponds to gene families present in at least 99% of the genomes of the sample. This provides some flexibility to account for sequencing or assembling artifacts and to account for the odd genome that may have recently lost a few core genes. The pan-genome families were classified as part of the *persistent* genome (3%), *singletons* (44%, present in a single genome), or *accessory* genome (the remaining) (Fig 1B, S2 Fig). The persistent gene families are a tiny fraction of the pan-genome, but account for half of the average genome (Fig 1B). They were used to build a robust phylogeny of the species (S3 Fig), which was rooted using genomes from other species in the genus (S4 Fig). In contrast, singletons are almost half of the gene families of the pan-genome, but less than 1% of the average genome. As a consequence, the pan-genome is open, as measured by the fit to a Heaps’ law model [46], and increases on average by ~26 protein coding genes with the inclusion of a new genome (S2 Fig). Singletons are smaller than the other genes and tend to be located at the edge of contigs (44%). Hence, some of these singletons may result from sequencing and assembly artifacts (S3 Text and S5 Fig). When all the singletons were excluded, the pan-genome still remained open (S2 Fig).

To obtain a better understanding of the functional classifications of genes in the pan-genome, we annotated them using the EggNOG categories (Fig 1C). As expected, the persistent genome over-represented typical housekeeping functions, whereas the accessory genome over-represented cell motility, intracellular trafficking and secretion, carbohydrate transport and metabolism and secondary metabolism. Singletons over-represented defense systems and genes related with the cell envelope. Most singletons (80%) and accessory (74%) gene families, but also a surprisingly high number of persistent gene families (24%), lacked a clear functional assignment as given by the EggNOG database [47] (Fig 1D). Hence, we are still ignorant of the function, or even the existence, of many genes of the species.

### Very rapid initial divergence of gene repertoires becomes linear with time

**Fig 2:**
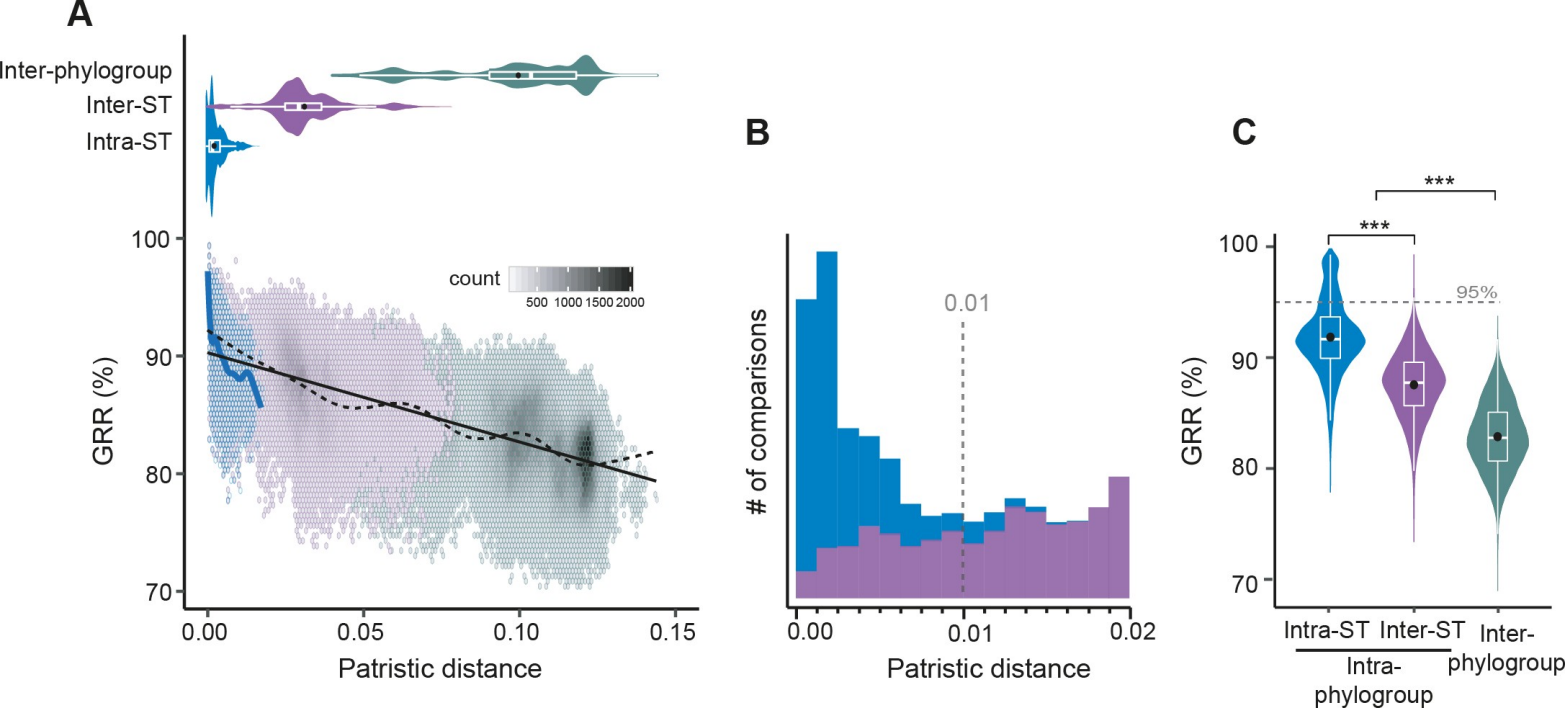
Evolution of Gene Repertoire Relatedness (GRR) with time. **A.** [Top] Violin plots of the patristic distance computed between pairs of intra-ST (in blue), inter-ST (in purple), and inter-phylogroup (in water green) genomes. [Bottom] Association between GRR and the patristic distance across pairs of genomes. Due to the large number of comparisons (points), we divided the plot area in regular hexagons. Color intensity is proportional to the number of cases (count) in each hexagon. The linear fit (black solid line, linear model (lm)) was computed for the entire dataset (1,294 genomes, Y = 90.2–75.7*X, R^2^ = 0.49, P<10^−4^). The spline fit (generalized additive model (gam)) was computed for the whole (in black dashed line) or the intra-ST (in blue solid line) comparisons. There was a significant negative correlation between GRR and the patristic distance (Spearman’s rho = -0.67, P<10^−4^). **B.** Stacked bar plot of the number of intra-ST (in blue) and inter-ST (in purple) comparisons at short evolutionary scales. **C.** Violin plots of the intra-ST, inter-ST and inter-phylogroup GRR (%). (A-B-C*)* All the distributions were significantly different (Wilcoxon test, P<10^−4^), the same color code was used and described in panel A.

Traditional epidemiological studies of *E*. *coli* focused on multilocus sequence types (ST) and/or the O-serogroups and H-types (the O:H combination corresponding to the serotype). These epidemiological units regroup strains in terms of sequence similarity in a few persistent genes (ST) or in key traits related to the cell envelope (the LPS structure for the O-group and the flagellum for the H-type). However, it is unclear if these types systematically regroup strains with similar gene repertoires. We identified 442 distinct STs, of which 61% are represented by a single strain. A few STs are very abundant in our dataset: 20 include more than 10 genomes each and encompass 40% of the dataset. STs are usually regarded as very recently diverged strains. Indeed, the intra-ST genetic distances are 10-times smaller those between the other pairs of genomes (0.003 vs. 0.03, Fig 2A). Yet, 6% of intra-ST comparisons have more than 0.01 substitutions per position showing extensive genetic diversity at the genome level (Fig 2B). Some O-groups are abundant, e.g., O8, O2 and O1 (each present in >50 genomes) but almost half of the groups occur in a single genome and 43% of the strains could not be assigned an O-group (even when the *wzm*/*wzt* and *wzx*/*wzy* genes were present). In contrast, most H-types were previously known (87%). We found 311 O:H serotypes among the 726 typeable genomes. Of these, 64% are present in only one genome, 17% are in multiple STs and 7% in multiple phylogroups (e.g. O8:H10). Conversely, half of the 95 STs with more than one genome have multiple O:H combinations, e.g. ST10 has 24. These results confirm that surface antigens and their combinations change quickly and are homoplasic. They also show significant sequence divergence in persistent genes within STs.

We then aimed at assessing if genomes within STs also show extensive variation of gene repertoires. For this, we computed the gene repertoire relatedness (GRR) between genomes (see Methods). Genes from the same gene family are on average 98.3% identical (S2 Fig). Since the threshold to be part of the family is 80% identity, rapid sequence evolution will very rarely lead a gene to be classed apart from its orthologs. As a result, variations in GRR result from gain and loss of genes, not sequence divergence. The GRR values decrease very rapidly with patristic distance (the sum of branch lengths in the path between two genomes in the phylogenetic tree) for closely related strains, as revealed by spline fits (Fig 2A). Similar results were observed when removing singletons, which only account for on average 0.5% of the genes in genomes, suggesting that this result is not due to annotation or sequencing errors (S6 Fig). As a consequence, 85% of the intra-ST comparisons have a GRR lower than 95% (corresponding to ~235 gene differences per genome pair), and some as little as 77% (Fig 2C). These results reveal that even genomes of the same ST can differ substantially in terms of their gene repertoires.

To check if the dataset is representative of the species and can be used to assess its diversity, we compared it with the ECOR collection [48] and the complete genomes available in RefSeq (Materials). All datasets had similar nucleotide diversity (S7A Fig and S1 Table). Using rarefied datasets, to compare sets of same size, ours had the largest pan-genome, partly because of a larger number of singletons (S7 Fig). Our dataset also had the highest α-diversity for the three typing schemes (STs, O-groups, H-types, S1 Table). Since the gene repertoire diversity of *E*. *coli* in Australia is at least as high as that of ECOR and RefSeq, we studied the variation in gene repertoires beyond the intra-ST level. After the rapid initial drop in GRR described above, the values of this variable decrease linearly with phylogenetic distances (Fig 2A). The average values of GRR given by the regression vary between 90% for very close genomes and 80% for the most distant ones. The variance around the regression line is constant and a spline fit shows few deviations around the regression line. This is consistent with a model where initial divergence in gene repertoires is driven by rapid turnover of novel genes. After this initial process, divergence in gene repertoires increases linearly with patristic distance.

### Rates of gene repertoire diversification vary across phylogroups

**Fig 3:**
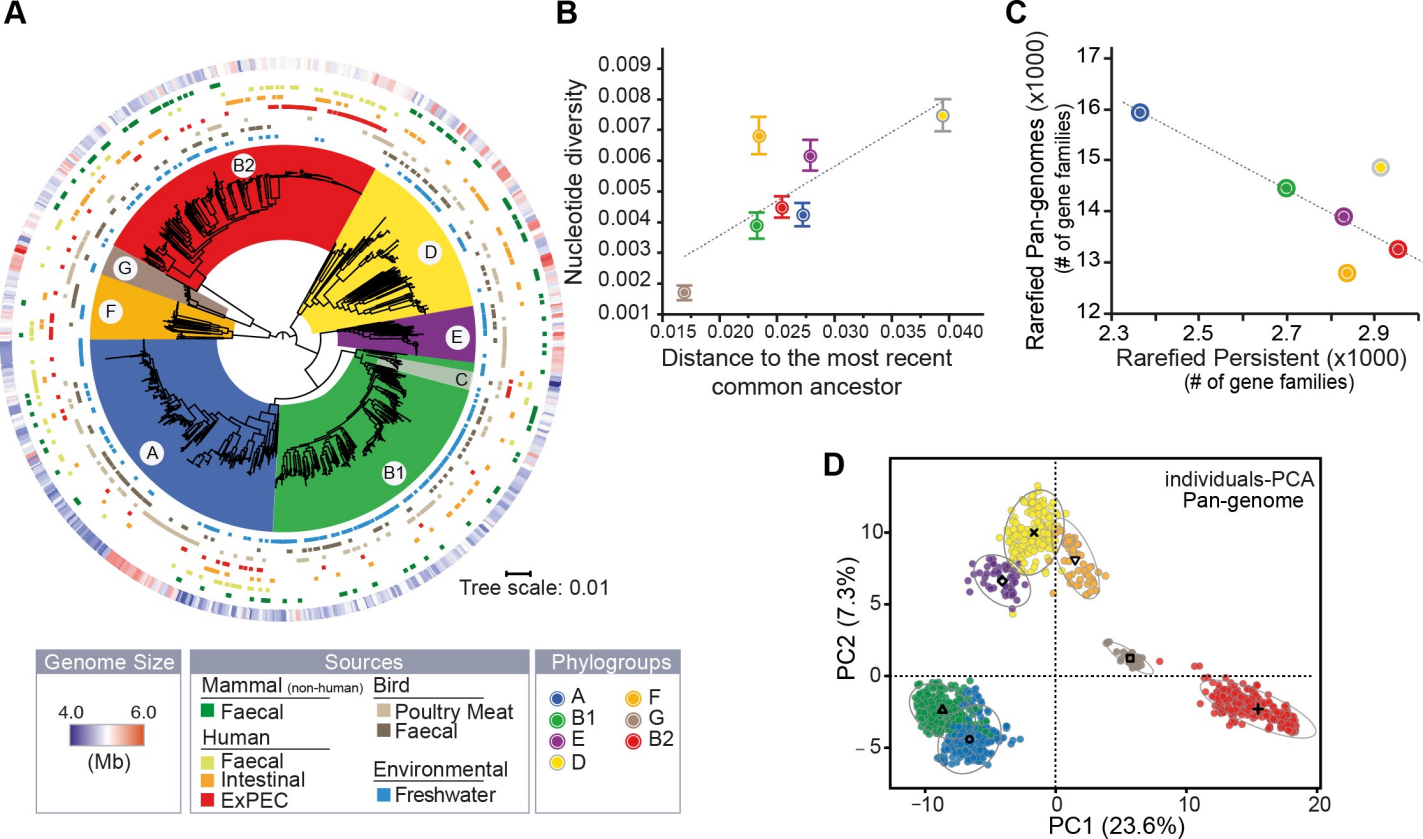
The genetic and ecological structure of Australian *E*. *coli* population. **A.** Phylogenetic tree of *E*. *coli* rooted using the genomes of other *Escherichia* (only shown in S4 Fig for clarity). From the inside to the outside: the 7 main phylogroups (arcs covering the tree), the source of each genome (seven rows), and the size of the genomes (outer row, see insert legend). **B.** Association between the nucleotide diversity per site (Pi, average and s.e) within phylogroup and their distance to their most recent common ancestor (MRCA). In each pylogroup, we averaged the nucleotide diversity (π) obtained for 112 core-genes, and the length branches (from tip-to-MRCA) of the species tree. **C.** Association between the rarefied pan- and persistent-genomes in each phylogroup. We used 1,000 permutations (genomes orderings) of 50 randomly selected genomes (rarefied datasets) to compute the pan- and the persistent-genomes in each phylogroup (ignoring the G group), and then averaged the results. **D.** Principal component analysis of the pan-genome (matrix of presence/absence of each gene family across genomes). Each dot corresponds to a genome in the two first principal components (PC). The ellipse (90%) and barycenter of each phylogroup are reported. The percentages in the axis labels correspond to the fraction of variation explained by the PC. All panels follow the color code of A.

We used the species phylogeny to study the associations between phylogroups and genetic diversity (Fig 3A). The tree showed seven main phylogenetic groups very clearly separated by nodes with 100% bootstrap support. The 17 phylogroup C strains were all included within the B1 phylogroup and were thus grouped with the latter in this study. For the rest, the analysis showed a good correspondence between the assignment into the known phylogroups—A, B1, B2, D, E, F, and G–and the different clades of the species tree. The tree splits the species initially in a clade with phylogroup B2, F and G on one side and the remaining on the other side. In line with the literature [40], four major phylogroups were very abundant—A (24% of the dataset), B1 (24%), B2 (25%) and D (14%)–whereas the others were rarer. The nucleotide diversity of the phylogroups is very dependent on their phylogenetic structure, since some clades have more closely related clusters of strains than others (S8 Fig). Nevertheless, nucleotide diversity, patristic distances, and Mash distances revealed similar trends: the phylogroup D exhibited the highest genetic diversity, followed by F, E, and then by the most abundant groups–A, B1 and B2 –which all have similar levels of diversity (S8 Fig). The phylogroup G was the least diverse, but it is also poorly represented in our dataset (33 genomes from three STs). Overall, genetic diversity is proportional to the depth of the phylogroup, i.e. the average tip-to-MRCA distance, except for phylogroup F which is more diverse than expected (Fig 3B). These results suggest that genetic diversity varies between phylogroups and that within phylogroups it is strongly affected by the time of divergence since the most recent common ancestor.

The sets of genomes of each phylogroup have large and open pan-genomes (S9 Fig and S2 Table). The sizes of these pan-genomes differ widely across phylogroups and are partly correlated to the number of genomes in the phylogroup, explaining why the phylogroup G has the smallest pan-genome (S9 Fig). To control for the effect of sample size, we computed pan-genomes from 1,000 random samples of 50 genomes for each phylogroup (ignoring the few strains of the G phylogroup, Fig 3C and S2 Table). This revealed larger pan-genomes for phylogroups A, D, and B1 followed by E, B2 and F. Intriguingly, the larger the pan-genome of a phylogroup, the smaller the fraction of its genes that are part of the persistent genome (Fig 3C). This suggests that differences of pan-genome sizes across phylogroups are caused by different rates of gene turnover, which seems to affect, at different extent, both genes present in most strains and genes present in very few.

To quantify the similarities in gene repertoires, we analyzed the GRR values between phylogroups. The smallest values were observed when comparing B2 strains with the rest (S10A Fig). Accordingly, a principal component analysis (PCA) of the presence/absence matrix of the pan-genome shows a first axis (accounting for 23.6% of the variance) clearly separating the B2 from the other phylogroups (Fig 3D). This shows that gene repertoires of B2 strains are the most distinct from the other major phylogroups. The large phylogroups A and B1 are very close in the GRR and in the PCA analyses, showing high similarity in terms of gene repertoires. Interestingly, the phylogroups D and F, which are not close in the species tree, cluster together in terms of gene repertoires. This may explain the conflicting results of our phylogenetic analysis, which places with high confidence the phylogroup D in the same partition of A and B1, and works based on ancestral gene repertoires that place them as a basal group in the tree (not far from F and G) [49]. Hence, phylogroups differ in terms of their gene repertoires and in their rates of genetic diversification, but while some are quite similar (A and B1), others (B2) stand aside from the remaining phylogroups.

### Mobile genetic elements drive rapid initial turnover of gene repertoires

**Fig 4:**
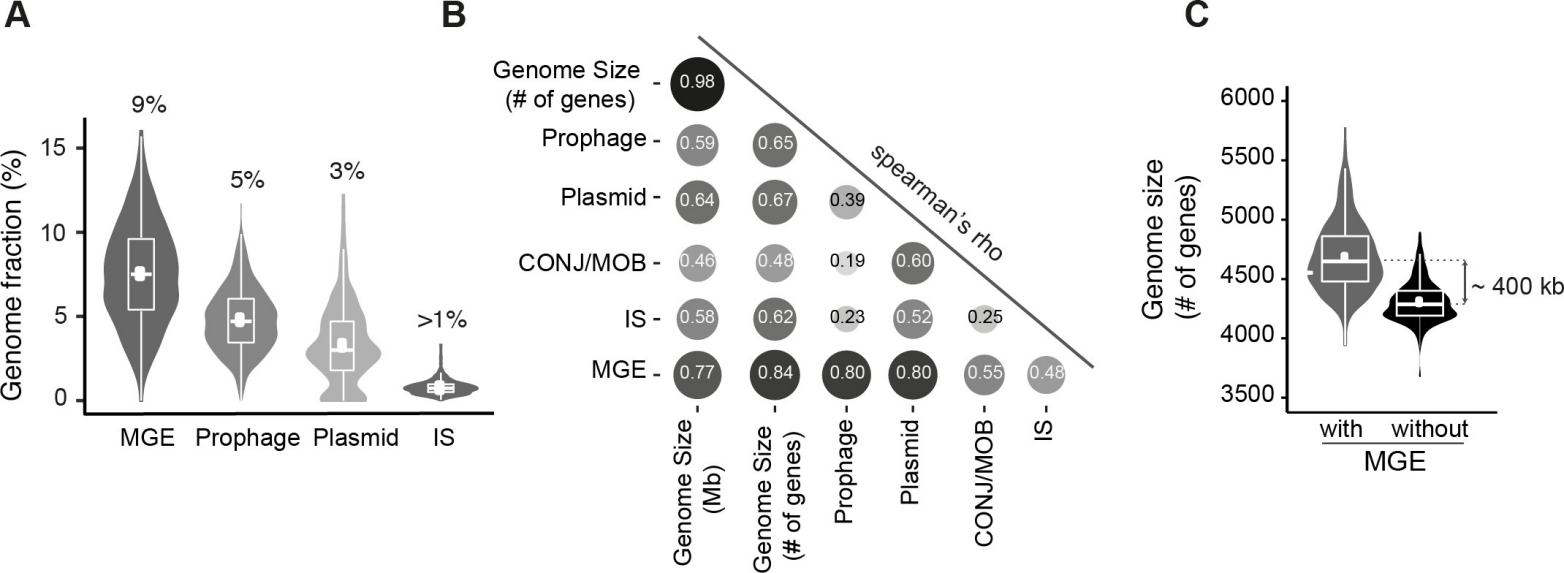
Frequency of mobile genetic elements (MGEs). **A.** Percentage of genes associated with MGEs per genome (sum in first graph). **B.** Spearman’s rank correlation matrix between the number of genes related to MGE and the genome size (in Mb and number of genes). The shades of the grayscale and the size of the circle are proportional to the correlation coefficients. All values are significantly positive (P<10^−4^). **C.** Differences in genome size when MGE genes are included or removed.

Different mechanisms can drive the rapid initial diversification of gene repertoires. Mobile genetic elements encoding the mechanisms for transmission between genomes (using virions or conjugation) or within genomes (insertion sequences, integron cassettes) are known to transfer at high rates and be rapidly lost [50–52]. We detected prophages using VirSorter [53], plasmids using PlaScope [54], and conjugative systems using ConjScan [55] (S11–S13 Figs). These analyses have the caveat that some mobile elements may be split in different contigs, resulting in missed and/or artificially split elements. This is more frequent in the case of plasmids, since they tend to have many repeated elements [56]. Only two genomes lacked identifiable prophages and only 9% lacked plasmid contigs. We identified 929 conjugative systems, with some genomes containing up to seven, most often of type MPF_F_, the type present in the F plasmid. On average, prophages accounted for 5% and plasmids for 3% of the genomes (Fig 4A). Together they account for more than a third of the pan-genomes of each phylogroup. We also searched for elements capable of mobilizing genes within genomes: Insertion Sequences, with ISfinder [57], and Integrons, with IntegronFinder [58]. Even if ISs are often lost during sequence assembly, some genomes had up to 152 identifiable ISs representing ~1% of the genome (Fig 4A and S13 Fig). A fourth of the ISs were in plasmids and very few were within prophages. We found integron integrases in 14% of the genomes, usually in a single copy. It is interesting to note that even if the frequency of each type of MGE varies across strains, each of them is strongly correlated with the frequency of the other elements (Fig 4B). Hence, the typical *E*. *coli* genome has at least one transposable element, a prophage and a plasmid, the key tools to move genes between and within genomes. This means that when genomes are enriched in one type of MGE, they tend to get simultaneously enriched in the remaining types of MGEs.

What is the effect of these MGEs in the dynamics of *E*. *coli* genomes? First, none of the MGEs gene families is present in more than 99% of the strains (i.e. none qualifies as persistent genes) at the species or at the phylogroup level. Instead, they are systematically at low frequency in the pan-genome, even at the phylogroup level. Hence, these genes rarely rise to high frequency in the species. Second, when we inferred the events of gene gain and loss in the species tree using Count (see Methods), we found that half of the recent gene acquisitions, i.e., those that took place at the level of the terminal branches of the species tree, were MGE genes. Conversely, the acquisitions at the terminal branches correspond to 40% of the MGE genes of the species. Third, the acquisition of MGEs affects the size of the genome. Those identified in this study account for ~8% of the genome size (Fig 4C and S14 Fig), and the number of genes associated with MGEs is strongly correlated with genome size for every type of element (Fig 4B). Fourth, MGEs increase the variability of genome sizes, since removing them decreases the coefficient of variation of the size of gene repertoires by 34% (expected increase of 4% under a Poisson model, Fig 4C). Fifth, the increase in variance in terms of genome size caused by MGEs is amplified by their rapid loss after acquisition (short persistence times in the genome). No MGE-associated gene family is sufficiently frequent to be part of the persistent genome, and most (85%) are present in less than 1% of the genomes. For example, 41% of the IS gene families are singletons (S14 Fig).

These results are consistent with the analysis of the variation in GRR with patristic distance, where some genes have extremely rapid turnover. Here we show that many of them are MGEs. The lack of fixation of MGE-associated genes suggests that the long-term cost of MGEs themselves is significant and/or their contribution to fitness is low (or temporary). But even if most genes associated with MGEs are eventually lost, their cargo genes may be adaptive, remain in the genomes for long periods and eventually become fixed. In conclusion, MGEs have a key role in the initial rapid turnover of genes in genomes because they are aquired at high rates, even if most of their genes are eventually lost.

### The smallest genomes have the highest gene turnover

**Fig 5:**
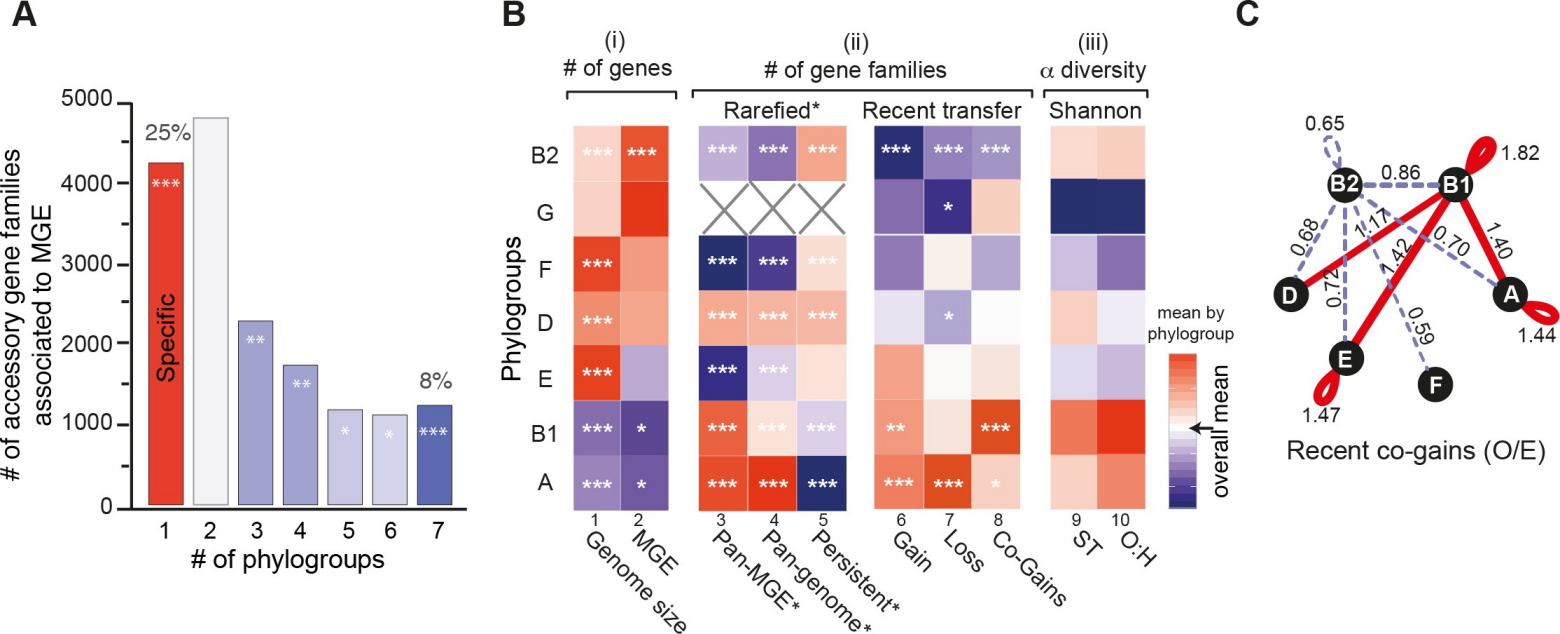
Genetic diversification across phylogroups. **A.** Number of accessory gene families associated to MGE present in one (i.e., phylogroup-specific) to seven phylogroups. The color code used corresponds to the Z-score obtained for the observed number (O) with respect to the expected distribution (E) (see Methods) for each case with a color code ranging from blue (under-representation) to red (over-representation). The level of significance was reported: |Z-score|: * ([1.96–2.58[), ** ([2.58–3.29[, ***([3.29). **B.** Heatmap where a cell represents the deviation (the difference) of the phylogroup to the rest. All values were standardized by column. The color code ranging from blue (lower) to red (higher), with white (overall mean). The level of significance of each ANOM test was reported: * (P<0.05), ** (P<0.01), *** (P<0.001). **C.** Network of recent co-occurence of gains (co-gains) of accessory genes within and between phylogroups. Nodes are phylogroups and edges the O/E ratio of the number of pairs of accessory genes (from the same gene family) acquired in the terminal branches of the tree. Only significant O/E values (and edges) are plotted (|Z-score|>1.96). Under-represented values are in dash blue and over-represented in red (see Methods).

Is the distribution of specific MGEs and their rates of transfer strongly associated with specific traits of genomes, like their phylogroup or isolation source? And if so, is this leading to preferential paths of gene transfer within the species? It has been suggested that homologous recombination is much rarer between than within phylogroups [18]. To test if this applies to the transfer of MGEs, we analyzed the distribution of the pan-genome gene families that are part of MGEs (excluding singletons, for the separate analysis of prophages and plasmids, see S15 Fig). There is a small but significant tendency of gene families of MGEs to cluster in a single phylogroup (Z-score>20, see Methods). However, 75% of the phage and plasmid gene families were found in more than one phylogroup and 8% were found in all phylogroups (Fig 5A). Hence, MGEs are key players in genome diversification at the micro-evolutionary scale. Above we showed that they were acquired independently multiple times and most of them have just arrived in their host genome. We now show that they are often transferred across phylogroups.

One might expect more genetic diversity in phylogroups with more MGEs and larger genomes. In apparent agreement with this hypothesis, genomes from phylogroups A and B1 are significantly smaller than the others (Fig 5B, col 1, ANOM tests, P<10^−3^) and have fewer MGE-associated genes (Fig 5B, col 2, ANOM tests, P<0.05). However, these phylogroups also have the largest diversity of gene families associated to MGEs (Fig 5B, col 3, in both the full and rarefied datasets, both ANOM tests, P<10^−3^), *i*.*e*. they encode fewer but more diverse MGEs. Furthermore, the phylogroups A and B1, in spite of having among the most recent common ancestors of the phylogroups (Fig 3B), have the largest pan-genomes, the smallest persistent genomes, and the largest diversity of STs, and serotypes (Fig 5B, in both the full and rarefied datasets, cols 4,5,9,10, ANOM tests, P<10^−3^). This intriguing pattern suggests that the smallest genomes have the highest turnover of genes, not the lower rates of transfer. To test this hypothesis, we took the quantification of gene gains and losses at the terminal branches of the species tree, computed with Count (see above), and computed the number of these events per phylogroup. We found that phylogroups A and B1 have the highest number of gene gains and losses per terminal branch (Fig 5B, cols 6–7). Hence, these phylogroups have the smallest genomes but the most frequent events of gene gain and loss.

To study recent gene flow between different phylogroups, we took the genes inferred to be acquired in the terminal branches of the species trees. Among these recently acquired genes we selected the gene pairs from the same gene family (co-gains) that were from the same phylogroup (Fig 5B, col 8) and those corresponding to recent acquisition of the same gene family in two different phylogroups (see Methods, Fig 5C). The results were represented as a graph where the edges represent significantly fewer (dashed lines) or higher (solid lines) number of co-gains than expected by chance. We found that phylogroup B1 has significantly more co-gains of genes with other phylogroups than expected, while the inverse was observed for phylogroup B2. We reached similar results when considering only the co-gains associated with MGEs (S16 Fig). These results are consistent with the separation of the B2 phylogroup from the others in the PCA analysis (Fig 3D). They show that such separation is due to lower rates of transfer in B2, which leads to fewer co-gains within the phylogroup and between this and the other phylogroups. In summary, phylogroups differ in terms of their genome size and in their rates of genetic diversification, the two traits being inversely correlated within the species.

### Not everything is abundant everywhere: the interplay between phylogroups and sources in genetic diversification

**Fig 6:**
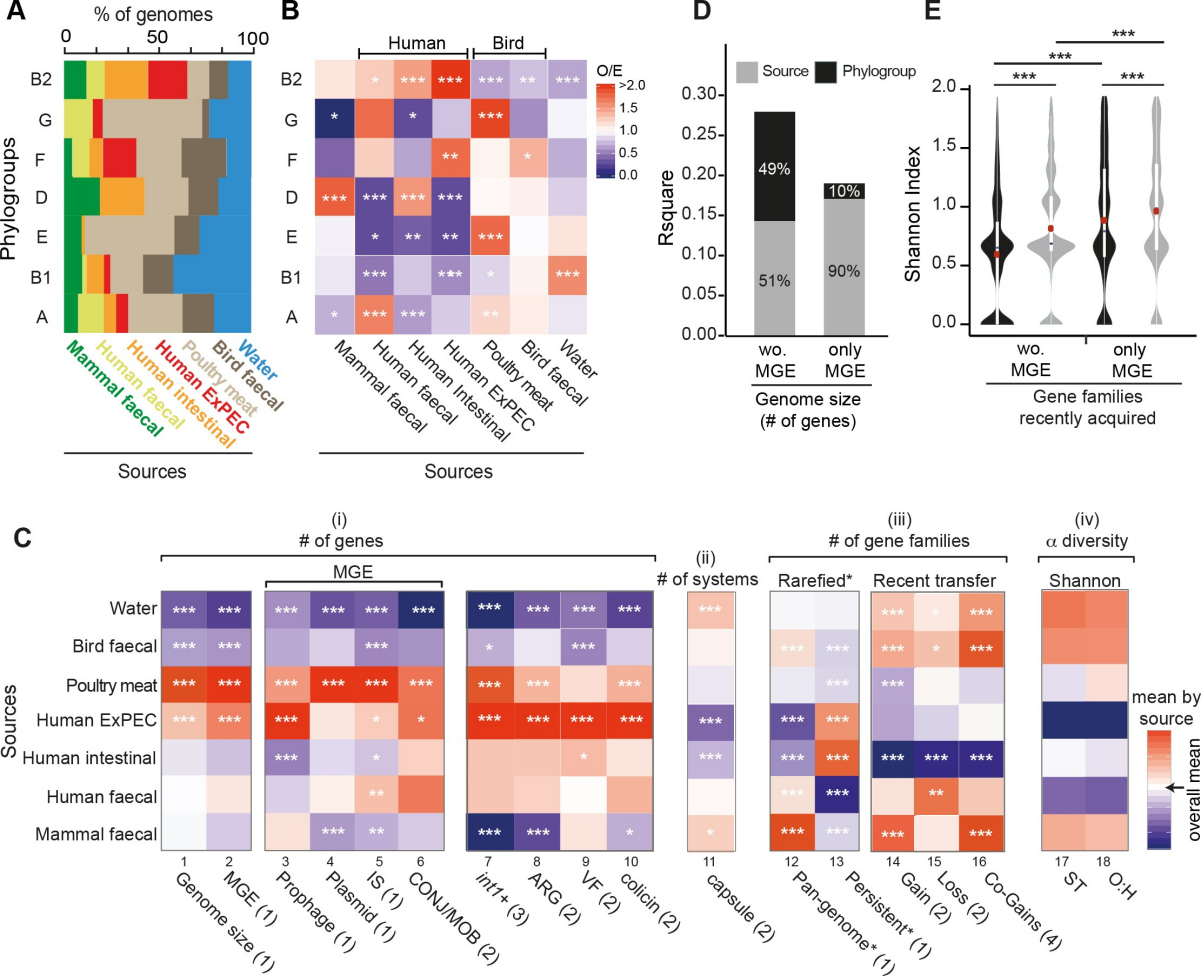
Genetic diversification across sources. **A.** Distributions of the sources in each phylogroup. **B.** Association between phylogroups and sources. The ratio of the number of observed (O) genomes divided by the expected (E) number was reported for all comparisons with a color code ranging from blue (under-representation) to red (over-representation) (Fisher’s exact tests performed on each 2*2 contingency table). **C.** Heatmap showing the associations between isolation sources and a number of traits. Each cell indicates the deviation (the difference) to the overall mean (in white). All values were standardized by column. Tests: standard ANOM (1), non-parametric ANOM tests (2, in presence of deviations from Gaussian distributions), ANOM for proportions (3). We represented the (O/E) ratio of the co-occurrence of gene pairs recently acquired (Co-gains) in each phylogroup with the same color code as in panel B (4). **D.** Contribution of each variable (phylogoup and source) to the variance explained by the stepwise multiple regressions of genome size (for the component of MGEs or the remaining genome) on phylogroup and the isolation source. **E.** Differences in diversity of gene families recently acquired across phylogroups (in black) and sources (in grey) for gene families associated to MGE or the remaining gene families (Wilcoxon tests, red dots (means)). In all panels: the level of significance of each test was reported: * (P<0.05), ** (P<0.01), *** (P<0.001).

Frequent horizontal transfer across phylogroups could result in adaptation being independent of the strain genetic background. While we observed that strains from all phylogroups could be isolated in all different sources (Fig 6A), different phylogroups are typically over-represented in some sources and rare in others (Fig 6B). These observations match previous studies [3], and show an association between the phylogenetic structure of populations and the natural habitats of the strains.

How much of the variability in genome size is explained by the source of isolation of the strains? Genome sizes vary significantly across isolation sources. Strains isolated from poultry meat had the largest average genomes, followed by human ExPEC strains. In contrast, strains from wild birds’ feces and freshwater had the smallest genomes (Fig 3A and Fig 6C, col 1, ANOM tests, P<10^−3^). We showed above that genome size also varies across phylogroups. To understand the relative role of the two variables, isolation source and phylogroup, we made two complementary analyses. First, we compared the genome size of strains from different sources within each phylogroup. Even if the statistical power was sometimes low, this revealed trends similar to the ones observed across phylogroups (S17 Fig). Second, we used stepwise multiple regressions to assess the effects of phylogroup and the strains’ source on its genome size. Both variables contributed significantly, and in almost equal parts, to the statistical model and together explained 36% of the variance (R^2^ = 0.36; P<10^−4^, S3 Table). We found similar results after removing MGE-associated genes (Fig 6D and S4 Table). We conclude that both isolation source and phylogroup are equally associated with genome size.

Adaptation to a habitat depends on HGT, which is driven by MGEs. This led us to study the distribution of MGEs in relation to isolation sources. There are fewer MGE genes in strains isolated from freshwater and wild birds’ feces, which have smaller genome sizes, and more in strains from human ExPEC and poultry meat (Fig 6C, col 2, ANOM tests, P<10^−3^, and S5 Table). We observed similar trends within each phylogroup even if the statistical power was low (S17 Fig). The analysis of the relative contribution of phylogroups and isolation sources to the number of MGE genes showed that the source of the strain accounted for the vast majority of the explained variance (90%, full model: R^2^ = 0.19; P<10^−4^, Fig 6D and S6 Table). Accordingly, the number of MGE gene families present in a single source of isolation was higher than expected (Z-score >17, S15 Fig), and nearly one third of these were observed in multiple phylogroups. To quantify this trend, we counted recent independent gains (co-gains, see definition above) of the same gene family (see Methods). This was done for pairs of genomes within the same source and between different sources. The analysis revealed that co-gains were more frequent than expected within the same isolation source. (Fig 6C, col 16, see Methods). These results suggest that the contribution of MGEs to genome size is primarily driven by the source of the isolate rather than phylogroup membership.

The previous result could arise from preferential co-gains of MGEs in an isolation source relative to a phylogroup, i.e. to frequent transfer of a few MGEs in the multiple isolates from the same type of source. To test this hypothesis, we used the results from Count and built a matrix where for each gene family we indicate the acquisition or not of a gene in each of the terminal branches of the phylogenetic tree. We then compared the clustering of these recent acquisitions by phylogroup and by isolation source using Shannon indexes (see Methods). If the hypothesis is correct, we expected higher clustering (lower diversity) across sources than across phylogroups. We observed slightly higher clustering across phylogroups than across sources, both for MGE and for the other genes (Fig 6E). We conclude that the contribution of MGEs to genome size depends largely on the isolation source but that this does not reflect systematic gains of the same MGE genes in the same source. Instead, the higher frequency of MGEs in genomes of certain sources may result from higher density of MGEs in those habitats (higher infection rates), or from higher probability of acquiring MGEs with adaptive traits at certain sources (higher selection rates).

It is tempting to speculate that the association between the number of MGE genes and isolation sources reflects selection for the acquisition of locally adaptive functions that are transferred by these MGEs. To test this, we searched for the presence of a trait—antibiotic resistance–that has become adaptive only recently and that is frequently transferred by MGEs. We searched for antibiotic resistance genes (ARGs) in our dataset using the reference databases. Many of these ARGs were in integrons (~3 per integron), which is well documented [59], and genomes carrying integrons had more ARGs than the others (Wilcoxon test, P<10^−4^, S18 Fig). Expectedly, integrons and ARGs were more prevalent in human ExPEC and in poultry meat isolates (Fig 6C, cols 7–8) and S5 Table). Similar results were observed in the analyses at the level of each phylogroup (S18 Fig). The clear association of integrons and ARGs with human (or domesticated animals) isolates of *E*. *coli* independently of the phylogroups’ genetic background reinforces the idea that source-specific MGEs provide locally adaptive traits.

### Functional differences across phylogroups and isolation sources

**Fig 7:**
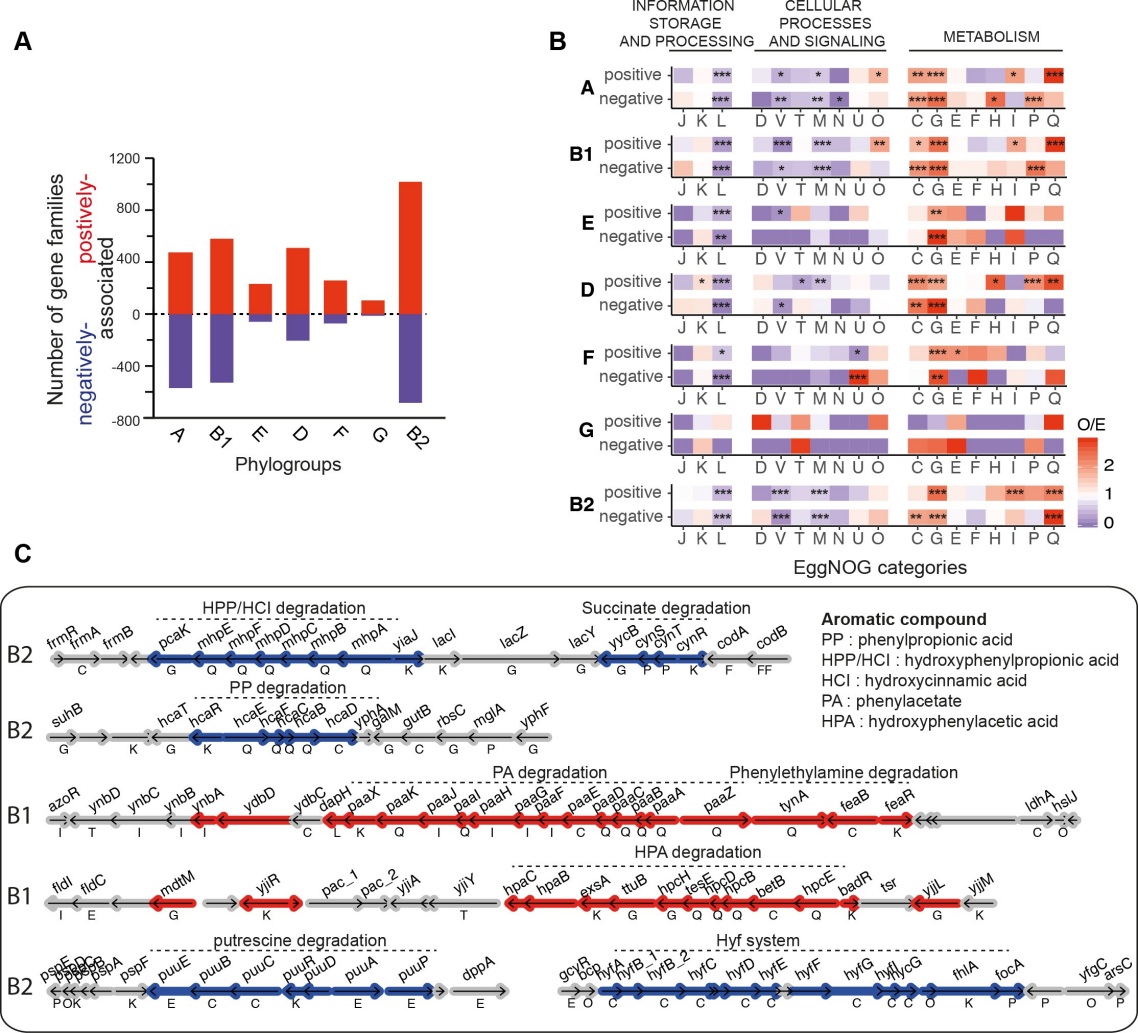
Genetic determinants of each phylogroup. **A.** Number of gene families positively (in red) and negatively (in blue) associated with each phylogroup. Altogether, they represent 7% of the accessory gene families of the dataset (note that some gene families are associated with several phylogroups). **B**. Observed/expected (O/E) ratios of non-supervised orthologous groups (NOGs, shown as capitalized letters, same code as shown in Fig 1C) in the positively or negatively associated gene families. For example, in phylogroup A there is an over-represents of positive associations in class Q, whereas in class L for the same phylogroup A there is under-represention for both positive and negative associations. The ratio (O/E) was reported for all comparisons with a color code ranging from blue (under-representation) to red (over-representation). The level of significance of each Fisher’s exact test was indicated (P> = 0.05 : ns; P<0.05 : *; P<0.01 : **; P<0.001 :***). It was performed on each 2*2 contingency table. Gene families lacking matches to the EggNOG functional categories (57%) were discarded. **C.** Genomic organization of some regions enriched in genes positively (in red) or negatively (in blue) associated with a phylogroup (indicated on the left). Genes shown in grey are not significantly associated. The name of the gene (when available) is shown above it, its EggNOG functional category (when known) below it.

Several of the previous results suggest an accumulation of adaptive genes as patristic distances increase. We used a gene-based GWAS to search for functions enriched in phylogroups or in isolation sources (see Methods). The first analysis revealed many gene families (2,754, S2 Dataset) positively and negatively associated with the phylogroups (Fig 7A). While in most cases these associations link a gene family to a phylogroup, the phylogroup A and B1, which are close in the phylogeny (Fig 3A) and in terms of gene repertoires (Fig 3D), have many associations in common (53%). The phylogroup with the largest number of associated genes is B2, which is also in accordance with the PCA analysis that revealed distinct gene repertoires in this phylogroup (Fig 3D). We characterized the functional categories of these associated gene families using EggNOG classification (as previously, S2 Dataset). In general, the categories over-represented are related to genes involved in metabolism (Fig 7B), which is in agreement with previous studies [60, 61].

The genes that were identified in the GWAS often concerned degradation processes, notably aromatic compound degradation (S2 Dataset) [62]. For example, PP (phenylpropionic acid) and HPP/HCI (hydroxyphenylpropionic and hydroxycinnamic acid) degradation pathways are negatively associated with B2 strains, while PA (phenylacetate acid) and HPA (hydroxyphenylacetic acid) degradation are positively associated with B1 strains (S2 Dataset, Fig 7C). These results are consistent with recent phenotypic tests (growth on specific substrates) [61]. Interestingly, B1 strains are positively associated with genes involved in rhamnose, sucrose, xylose, glycerate, and tartrate degradation pathways, while B2 are negatively associated with traits associated with plant colonization such as the Hyf system (involved in control and pH control), melibiose, cyanate, putrescine, and D-malate degradation pathways (S2 Dataset, Fig 7C). These pathways are involved in alternate carbon source metabolism, and may reflect functional adaptations to different nutritional environments, as proposed previously [63]. These results suggest that B1 strains, contrary to B2, tend to carry traits facilitating adaptation to environmental niches, such as soil and water (where aromatic compounds are highly abundant) or to colonize plants, as previously suggested [64].

**Fig 8:**
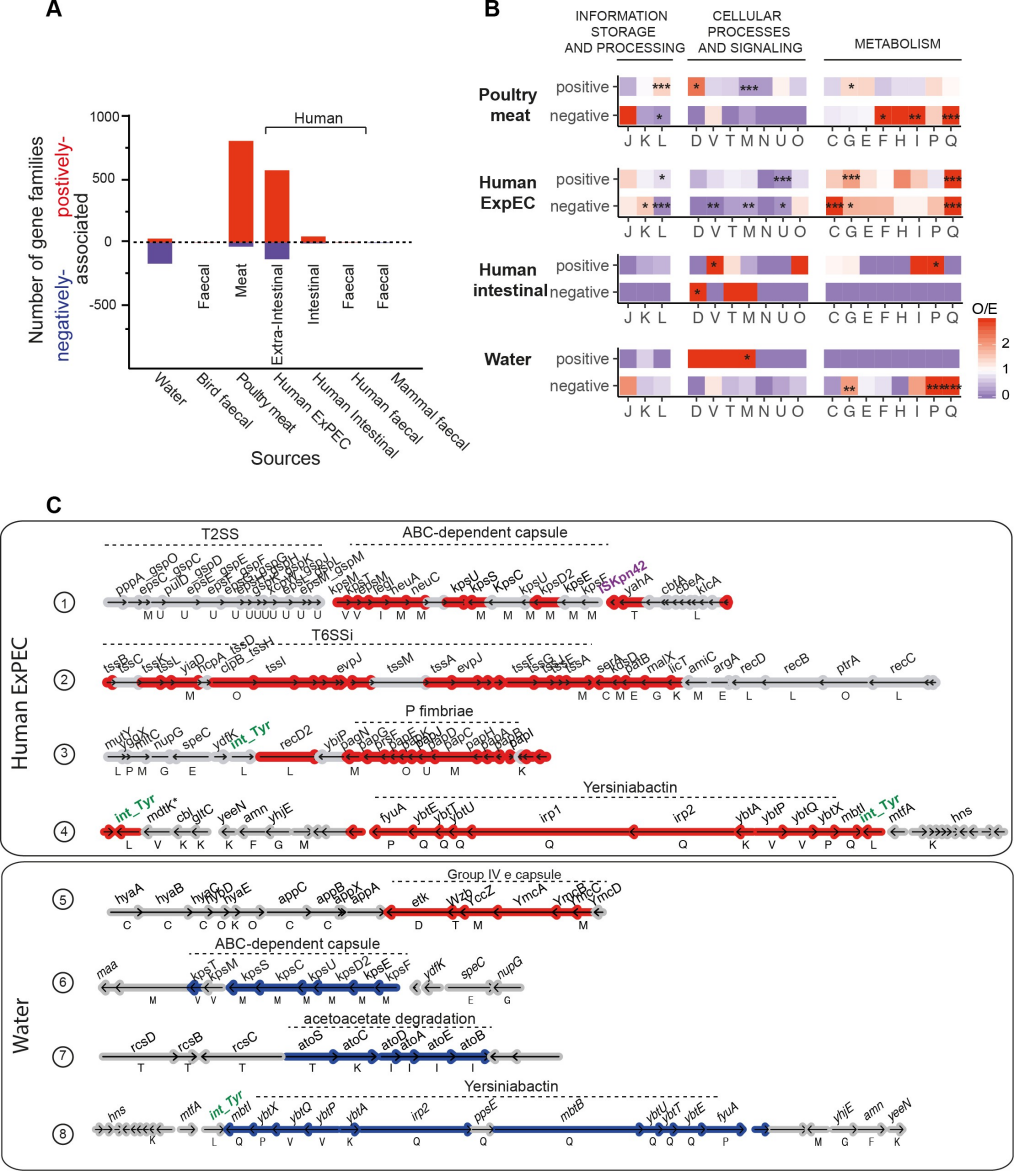
Genetic determinants of each isolation source. **A.** Number of gene families positively (in red) and negatively (in blue) associated with each source. **B**. Observed/expected (O/E) ratios of non-supervised orthologous groups (NOGs, shown as capitalized letters, same as in Fig 1C) in the positively or negatively associated gene families. The ratio (O/E) was reported for all comparisons with a color code ranging from blue (under-representation) to red (over-representation). The level of significance of each Fisher’s exact test was indicated (P> = 0.05: ns; P<0.05: *; P<0.01: **; P<0.001: ***). It was performed on each 2*2 contingency table. Only gene families with known functions were considered in this analysis. Gene families lacking matches to the EggNOG functional categories were discarded.**C**. *Genomic organization of regions enriched in genes strongly positively (in red) or negatively (in blue) associated with a source*. *Genes shown in grey are not significantly associated*. *The name of the gene (when available) is shown above it*, *its functional category (when known) below it*.

The same analysis made at the level of the isolation sources revealed fewer genes (Fig 8A). The different fecal isolates almost lacked associated genes, presumably because this is the most typical and the ancestral environment of the species and it may have adapted to it for a long time. We therefore focused our analysis on genes involved in virulence. The analysis of human ExPEC isolates revealed many associated genes (S2 Dataset), including well-known virulence factors such as ABC-dependent capsule systems, the motility repressor *papX*, the P fimbriae, yersiniabactin, colibactin and multiple type 5a protein secretion systems (Fig 8C). To complement this analysis, we searched specifically for known virulence factors from VFDB [65]. Indeed, they are more prevalent in human strains, and especially in ExPEC isolates (ANOM test, P<10^−3^), while being rare in strains isolated from freshwater and wild birds’ feces (ANOM test, P<10^−3^, Fig 6C, col 9). While these virulence factors are more concentrated in phylogroups B2, D, E and F (ANOM test, P<10^−2^) as previously shown [37], the trends regarding isolation sources are conserved within each phylogroup (S19 Fig). In particular, within phylogroup B2, only human strains have a significantly higher average number of virulence factors (S19 Fig) as previously suggested [26].

While virulence factors were associated with human isolates, we oberved associations between certain isolate sources and mechanisms used in antagonistic interactions with other bacteria. This includes overpresentation of type VI secretion systems (T6SSi) in ExPEC, type 5b secretion systems (often associated with contact-dependent inhibition) in poultry meat isolates, and bacteriocins in several isolation sources (S2 Dataset). To detail these results, we searched specifically for colicin gene clusters [66], using BAGEL3 [67] (some of which are also included in VFDB). We found from an average of 2.8 genes in B2 strains to 0.4 in B1 strains. Interestingly, the water isolates have the fewest colicin genes, presumably because free diffusion of these proteins in water makes them inefficient tools of bacterial competition (Fig 6C, col 10 and S19 Fig). Thus, local adaptations resulting from the acquisition of novel genes by HGT, involving antagonistic interactions with other bacteria are associated preferably with certain phylogroups.

### *E*. *coli* from freshwater are different

*E*. *coli* has usually been regarded as a contaminant from animal, mostly human, sources and used to test water quality. Yet, recent data suggests that some strains could inhabit aquatic environments [68]. Given the contrast between the primary and secondary habitats of *E*. *coli*, respectively guts of endotherms and aquatic environments, this would imply marked differences between the 285 freshwater strains and the others. Indeed, our results show that these strains are systematically different. They are over-represented in phylogroup B1 (43%), a phylogroup under-represented in all other sources of isolation (Fig 6A and Fig 6B). On the other hand, they are under-represented in B2 (13%), a phylogroup over-represented in strains isolated from humans (this study) and other mammals [2]. The genome size of freshwater strains’ is the smallest among all groups of isolates and across phylogroups (Fig 6C, col 1, S17 Fig). Importantly, these strains show average pan-genome sizes in the rarefied dataset, suggesting that adaptation is not exclusively due to genome reduction (Fig 6C, col 12). This is also supported by the high number of gains and losses observed (Fig 6C, cols 14,15), although these genomes have the fewest MGEs and often lack plasmids (Fig 6C, cols 2–6). Consistent with adaptation to this habitat, they have the smallest number of antibiotic resistance genes, virulence factors, and bacteriocins (Fig 6C, cols 7–10, S18 and S19 Figs). In contrast, these strains show the highest diversity of STs and O:H serotypes (Fig 6C, cols 17,18, and S5 Table), and the highest number of capsule systems (Fig 6C, col 12, S20 Fig).

The extreme genomic traits of isolates from water strongly suggest they are not the result of recent fecal contamination from other sources. Instead, they strongly suggest that these strains have changed to adapt to water environments. This change seems to have involved the loss of many genes, and this is apparent from the GWAS analysis, which shows many more negative than positive associations with this isolation source (contrary to all the others) (Fig 8A). Many of them correspond to the virulence factors described above (Fig 8C). The few gene families positively associated with freshwater are over-represented in the EggNOG category M (cell envelope, Fig 8B). Many of these correspond to genes encoding the Group IVe capsular genes (Fig 8C), which contrasts with ABC-dependent capsules that are positively associated with Human ExPEC strains (S2 Dataset). Capsules have been proposed to allow cells to withstand biotic and abiotic challenges, and these results suggest that they are an important component of *E*. *coli* adaptation to freshwater environments. Overall, these results show that *E*. *coli* in these environments endured some horizontal gene transfer and important genome streamlining, i.e. a high turnover of gene repertoires that resulted in genomes smaller than the average carrying a few specific adaptations to the environment.

## Discussion

Many of the recent advances in the understanding of *E*. *coli* evolution focused on clinical isolates and placed a lot of emphasis on virulence and antibiotic resistance in a few clinically important lineages [69–74]. Yet, most strains of the species are commensal. Hence, most of the evolution of the species takes place in biotic contexts not associated with pathogenesis. Furthermore, while a lot of attention has been given to the rates of homologous recombination in core genes, it is now clear that the acquisition of novel genes drives the evolution of virulence [12, 42, 75, 76] and antibiotic resistance [77–79] in pathogenic strains as well as that of many other traits in commensal strains [12]. For example, MGEs were recently shown to be more important than point mutations for the colonization of the mouse gut by *E*. *coli* commensals [80]. Here, we aimed at providing a global picture of the evolution of the *E*. *coli* genomes with an emphasis on the variation of gene repertoires in strains from a variety of sources (environmental and geographic) across a single continent. This allowed us to study the joint effect of population structure and habitat on the variation of gene repertoires. Our study focused on *E*. *coli* isolates from Australia, but its genetic diversity was higher or comparable to other worldwide genome datasets, and its population structure was consistent with previous works [16, 40, 81]. This indicates that what we have observed is likely to be representative of the species as a whole. It also confirms previous reports of the large genetic diversity of the species and of the planetary circulation of all major lineages [39, 45, 82]. Finally, the functional annotation of the pan-genome shows that in spite of over 375,000 papers citing *E*. *coli* in PubMed in 2019, we are still far from having discovered the full genetic diversity of *E*. *coli* and from knowing the function of many of its most frequent gene families.

We started our study by quantifying gene repertoire diversification, which we found to follow a two-step dynamic. The very rapid initial diversification, where GRR quickly decreases to ~90%, implicates substantial heterogeneity in terms of gene repertoires for strains that are from the same sequence type and are almost identical in the sequence of persistent genes. Some of the rapid initial divergence of GRR may be due to genome sequencing or assembling artifacts producing singletons and thus inflating pan-genomes. Yet, we have annotated all genomes in the same way. We also confirmed key results by excluding singletons, by showing that singletons represent only ~0.5% of a typical genome, and that many of them have homologs in the databases. The frequency of singletons is only weakly correlated with the number of contigs in draft assemblies, a further sign that they are not just caused by sequencing or assembly issues (S3 Text). Furthermore, our analysis of ancestral genomes showed that a large fraction of well-known MGEs, including phages, ISs and plasmids, were acquired very recently (inferred acquisition at the terminal branches of the phylogenetic tree). Some of these are singletons, whereas others are present across a few genomes of many phylogroups. They contribute directly to the very rapid divergence of gene repertoires between separating lineages. Hence, we do not think that technical issues alone explain the existence of rapid gene repertoire differentiation between recently divergent strains. This raises the question of how much these processes reflect natural selection on incoming genes or high rates of gene loss by drift.

Previous population genetics models applied to other clades observed the existence of genes that have rapid turnover in genomes, i.e. that are rapidly lost after being acquired [83, 84]. Our results show that frequent acquisition of MGEs drives rapid diversification of gene repertoires even between strains that are almost indistinguishable by classical typing schemes. In the present context, this suggests that either many integrations of genetic material are deleterious and get rapidly purged by natural selection or that they are of no lasting adaptive value and get rapidly deleted by genetic drift. The first hypothesis is consistent with the fitness costs associated with the acquisition of many MGEs [85–87], with our observation that most MGEs present in a genome were very recently acquired, and with the abovementioned rapid loss of GRR for small patristic distances. The second hypothesis is consistent with previous works suggesting the existence of mechanistic biases towards gene deletion in bacteria that quickly remove genes without adaptive value from the genome [88, 89]. It is also consistent with the observation that some classes of functions, like defense systems [90] or specific components of the cell envelope [91], are subject to fluctuating selection dynamics and become neutral or slightly deleterious (because costly) after a short period where they are selected for.

After the initial period of rapid GRR decrease with phylogenetic distance, GRR decreases linearly with divergence time, a trend that was not quite clear when we first analyzed this question a decade ago with a much smaller set of genomes [42]. Importantly, this linear decay is not suggestive of the existence of a point beyond which relatedness and gene flow change abruptly. Hence, these results do not suggest incipient sexual isolation within the species from the point of view of horizontal gene transfer. This is confirmed by our analysis that some MGEs are present in many phylogroups and by the finding that many gene families of the pan-genome were recently acquired independently by distantly related strains. An interesting feature of the comparisons of GRR in function of phylogenetic distances is the large variance around the regression line. This variance may result from very different processes. One of them may be preferential transfer of genes across strains within the same habitat, as observed for the isolates from the same type of source in this work. This type of transfer will lead to pairs of strains with more similar gene repertoires than expected given their patristic distance. Conversely, bacteria shifting from one habitat to another may endure an acceleration of their divergence in terms of gene repertoires. This will be a consequence of selection for different traits, acquired by HGT, and of changes in its preferential gene flow towards strains from the novel habitat.

The rapid evolution of gene repertoires by HGT is consistent with the observation that plasmids, prophages and ISs are almost ubiquitous among *E*. *coli*. These elements contribute significantly to genome size and even more to the variability of genome size across strains, which supports our previous results [51, 92]. While most MGEs are quickly lost from lineages, or drive the lineage extinct, the large influx of such elements can bring adaptive accessory traits such as antibiotic resistance genes [78] and virulence factors [93, 94]. They also pave the way for cooption processes [95]. The contribution of the MGE genes to genome size across the species is more strongly associated with the isolation source of the strains than with the phylogroup. However, the recent co-acquisition of MGEs by different strains is also associated with the phylogroup. This is consistent with a scenario where the abundance of MGEs in a genome is strongly dependent on the habitat, but their diversity also depends on the phylogroup. Since most MGE genes arrived in the genome very recently, this suggests that habitat exerts a strong constraint on the flow of gene exchanges across the species, in line with the view that bacteria exchange more genes with those they coinhabit with [96, 97].

The adaptive novel genetic information being acquired with MGEs must be integrated in the cell functioning. This need of favorable genetic backgrounds for certain local adaptation processes could explain the observed over-representation of some phylogroups in certain isolation sources. Virulence factors and antibiotic resistance genes provide relevant examples. In our dataset, the plasmids encoding virulence factors are often conjugative and should be able to circulate widely, but the virulent clones often concentrate in a few phylogroups. Selection for antibiotic resistance is expected to be higher in human-associated clones, and especially the virulent ones, because these are the most targeted in the clinic. Hence, they endure stronger selection to keep the ARGs arriving in MGEs. These causal links result in preferential associations of genetic backgrounds with virulence factors and ARGs, and therefore with the frequency of human isolates in a given phylogroup. It remains to be quantified the degree to which these trends are due to epistatic interactions between novel genes and the genetic background and to the availability of specific genes by horizontal transfer in certain sources. In conclusion, these results contribute to explain why epidemiological clones tend to emerge from specific phylogenetic groups even in the presence of massive horizontal gene transfer.

Genetic diversity, created by HGT, recombination, or mutation, affects a species' ability to adapt to novel ecological opportunities. The higher the diversity of gene repertoires in a population, the more likely that one of those genes will prove helpful in the face of environmental challenges such as antibiotics. We observed that the generalist phylogroups, such as A and B1, have larger pan-genomes than specialist phylogroups like B2. This was not expected based on their smaller genome sizes or the lower frequency of MGEs in their genomes. We propose that this reflects the high variability of the environments where they circulate—in terms of conditions, other strains and MGEs—and the associated diversity of local adaptation processes. Phylogroup B1, in particular, is associated with the presence of a number of metabolic traits suggesting interactions with plants. Phylogroup B2 strains, by comparison, have developed specific traits that may let them take advantage of some particular resources, e.g. they are better adapted to the mammal gut environment [2]. This has resulted in large genomes that are quite different from the other major phylogroups of *E*. *coli*, as revealed by the phylogeny of the species based on the polymorphism on persistent genes, the PCA analysis of the pan-genome matrix, the GWAS analysis, and the large number of MGEs identified in their genomes. Yet, they are overall more conserved (largest persistent-genome, smaller pan-genomes, fewer recent gene acquisitions). This may explain why it has been suggested that strains from phylogroups A, D and B1 derived from an ancestral B2-like genetic background. The conservation of a larger core genome is consistent with our quantification of genetic exchanges: B2 strains exchange less genetic material with strains from its own and from other phylogroups than the remaining large phylogroups. This has placed it apart in terms of gene repertoires and in terms of preferential habitats. Altogether, these results suggest that the habitat and the phylogenetic structure jointly determine the size of genomes. The results also suggest the hypothesis that the large genomes of some phylogroups, like B2, may be caused by a relative decrease in the rate of gene loss, and not necessarily by an increase in the rate of gene gain.

The integration of information on gene repertoires, population structure and isolation sources sheds some light on the origin of environmental strains. This is illustrated by the identification of genomic traits in freshwater *E*. *coli* isolates that are very different from the average traits of the species and that suggest adaptation of certain lineages to this environment. For bacteria, freshwater environments are much more nutrient poor than the guts of endotherms, and it's interesting to note that strains associated with this environment have more streamlined genomes. This may represent, at the micro-evolutionary scale, an adaptation similar to that observed in other bacteria adapted to poor nutrient environments that also have small genomes and few MGEs [98, 99]. These results are also consistent with recent studies showing that *E*. *coli* B1 strains can persist longer in water than strains of the other phylogroups, and that B1 strains isolated repeatedly in water often encode very few virulence factors and antibiotic resistance genes [7, 33, 34]. Interestingly these strains have been shown to be able to grow at low temperatures [7]. The prevalence of B1 isolates has been observed in other environmental samples, such as drinking water and plants [64]. The characteristics observed in freshwater isolates might be general to this environment, since they were observed in strains from the B1 and from other phylogroups (S16–S20 Figs). If some *E*. *coli* lineages are indeed adapted to freshwater this radically changes the range of environments from where they can acquire novel genes and the selection pressures that shape their subsequent fate. This finding also implies that environmental isolates are not necessarily the result of source-sink dynamics where *E*. *coli* strains evolve in relation to selection pressures linked to the host and environmental strains are just sinks where such strains find evolutionary dead-ends. Instead, the environment outside the host could have a significant impact on the evolution of *E*. *coli* subsequently colonizing human hosts.

## Materials and methods

### Strains

We used different collections of *E*. *coli* strains recovered in Australia between 1993 and 2015 (for a more detailed description, see S1 Text and S1 Dataset). The subset of strains selected for whole genome sequencing includes: (1) *faecal strains* isolated from various birds (N = 195 strains), non-human mammals (N = 135), and humans living in Australia (N = 93); (2) *clinical strains* isolated during intestinal biopsies of patients with inflammatory bowel disease (N = 172), or corresponding to human ExPEC strains collected from urine or blood (N = 112); (3) *poultry meat strains* isolated from chicken meat products from diverse supermarket chains and independent butcheries (N = 283); (4) and *freshwater strains* isolated from diverse locations across Australia (N = 285).

### Sequencing

Of the 1,304 isolates, 70 were sequenced at Broad institute using the Roche 454 GS FLX system (this was done 10 years ago, detailed in [100]), 70 were sequenced by GenoScreen (Lille, France) using the HiSeq2000 platform. The rest were sequenced at the Australian Cancer Research Foundation (ACRF) Biomolecular Resource Facility (BRF) of the Australian National University, using the Nextera XT sample preparation kit (Illumina) and the Illumina Miseq (paired-end sequencing), as detailed in [101].

### Assembling

Paired-end read files were processed and assembled with CLC Genomics Workbench v.9.5.3 (Illumina) using their *de novo* assembly algorithm with default parameters.

All genomes sequenced by the Broad institute were available into the NCBI Assembly (www.ncbi.nlm.nih.gov/assembly/) or SRA (www.ncbi.nlm.nih.gov/sra/) databases. While, the rest of the assemblies was deposited into the European Nucleotide Archive (PRJEB34791). The accession number of each genome is reported in S1 Dataset.

### Datasets

We used 4 datasets in this study. (1) The ***Australian dataset*** described above is the main (default) dataset. (2) ***RefSeq dataset***: We retrieved 370 *E*. *coli* complete genomes from GenBank Refseq (available in February 2018). (3) ***ECOR dataset***: We retrieved 72 draft genomes of the *E*. *coli* reference (ECOR) collection from DDBJ/ENA/GenBank [48]. Strains in this collection were isolated from diverse hosts and geographic locations and have been used for more than 30 years to represent the phylogenetic diversity of *E*. *coli* as they have been selected from over 2,600 natural isolates based on MLEE data [17]. (4) ***Outgroup dataset***: We retrieved 65 other closely related *Escherichia* genomes from ENA/GenBank and sequenced 21 others on the Illumina MiSeq platorm (assembled as described above). They belong to Clade I (N = 14), Clade II (N = 2), Clade III (N = 8), Clade IV (N = 2), Clade V (N = 14), *E*. *fergusonii* (N = 8) and *E*. *albertii* (N = 38) species. Only five of them were complete, others were draft genomes. In this study, these genomes (called hereafter *outgroup* genomes) were only used to root the Australian *E*. *coli* species tree. The general genomic features and the sequencing status of these 1,832 genomes are reported in S1 Dataset.

### Data formatting

In an attempt to overcome the bias from different annotations all genomes of the four datasets were annotated using Prokka v.1.11 [102] which provided consistency across the entire datasets (with hmmer v.3.1b1, aragorn v.1.2.36, barrnap v.0.4.2, minced v.0.1.6, blast+ v.2.2.28, prodigal v.2.60, infernal v.1.1, ncbi_toolbox v.20151127, and signalp v.4.0). We performed three quality controls on genomic sequences of Australian and outgroup datasets (see S2 Text). A total of 10 *E*. *coli* draft genomes and one genome from clade V failed at least one of these tests and were removed from further analysis, leading to a final dataset of 1,294 Australian *E*. *coli* genomes and 87 outgroup genomes. The main characteristics of each draft genome are reported in S1 Dataset.

*E*. *coli* typing. ***Phylogroup***. The phylogroup of each *E*. *coli* genome (from ECOR, RefSeq, and Australian datasets) was determined using the *in silico* ClermonTyping method [20]. ***Multilocus sequence typing*** (***MLST)*.** Sequence type (ST) was identified by the MLST scheme of Achtman [10] using mlst v.2.16.1 (https://github.com/tseemann/mlst). We assigned STs for a large majority of genomes, i.e., for 99%, 96% and 97% of the ECOR, RefSeq and Australian genomes resp. ***Serotype***. Serotype (O- and H-genotypes) was inferred with the EcOH database [103] using ABRicate v.0.8.10 (https://github.com/tseemann/abricate)). Currently there are 220 *E*. *coli* O-groups and 53 H-types described in this database. While 99% of Australian genomes had H-group assigned, only 57% had O-group assigned even if *wzm*/*wzt* and *wzx*/*wzy* genes are present. All these results are reported in S1 Dataset.

### Nucleotide diversity

The ***nucleotide diversity*** of the three datasets, *i*.*e*., ECOR, RefSeq and Australian, was computed from the multiple alignments of 112 core gene families present in all *E*. *coli* genomes of these three datasets, (see below), using the diversity.stats function from the *PopGenome* v.2.6.1 R package [104]. We also used these 112 core gene families to assess the nucleotide diversity for each phylogroup of the Australian dataset.

### ST and O:H diversity

The ***Shannon index*** was computed to assess the diversity of ST and O:H serotypes within each phylogroup and source. For this, we calculated their relative frequency in each group and then applied the function skbio.diversity.alpha_diversity from the *skbio*.*diversity* v.0.4.1 python package (http://scikit-bio.org/docs/0.4.1/diversity.html).

### Mash distances (M)

***Genome similarity*.** Due to the high cost of computing ANI [105] via whole-genome alignment, we estimated genome similarity calculating the pairwise Mash distance (M) between all Australian genomes using Mash v.2.0 [106]. Importantly, the correlation between the Mash distances (M) and ANI in the range of 90–100% has been shown to be very strong, with M ≈ 1-(ANI/100) [106]. All the resulting Mash distances between *E*. *coli* genomes are well below 0.05, in agreement with the assumption that they all belong to the same species. The median is 0.027 and the maximal value is 0.04 (S4 Fig). **Australian *E*. *coli* reference genomes**. The Mash distance was strongly correlated to the patristic distance in our dataset (spearman’s rho = 0.92, P<10^−4^). We used it to select 100 Australian *E*. *coli* strains representative of the species’ diversity (called hereafter *reference* genomes). Such *reference* genomes were used to root the Australian *E*. *coli* tree (to drastically reduce the computational time required to build the rooted tree). To select representative genomes, we performed a hierarchical WPGMA clustering from the Mash distance matrix computed with all Australian *E*. *coli* genomes, and then we cut it off to have only 100 clusters. In each of these clusters, the genome with the smallest L90 was selected. This *reference* dataset contained all the phylogroups and was composed of: 15-A, 10-B1, 13-E, 39-D, 11-F, 10-B2 and 2-G genomes.

### Identification of pan-genomes

Pan-genomes are the full complement of genes in the species (or dataset, or phylogroup) and were built by clustering homologous proteins into families. We determined the lists of putative homologs between pairs of genomes with MMseqs2 v.3.0 [107] by keeping only hits with at least 80% identity and an alignment covering at least 80% of both proteins. Homologs proteins were then clustered by single-linkage [108]. We computed independently the pan-genome of each dataset, *i*.*e*., ECOR, RefSeq, Australian and of the 87 outgroups with the 100 Australian *E*. *coli* reference genomes. Each pan-genome was then used to compute a matrix of presence-absence of gene families. Hence, gene copy number variations were not taken into account in this part of the study. The alpha exponent of Heap’s Law was used to infer whether a pan-genome is open or closed [46]. Thus, if α (alpha) < = 1, the pan-genome is open. In contrast, α (alpha) > 1 represents a closed pan-genome. This coefficient was computed using the *heaps* function of the *micropan* v.1.2 R package [109] with n.perm = 1000. Principal component decomposition of the Australian pan-genome, *i*.*e*, the matrix of presence-absence of protein families was computed using the *prcomp* function from the *stats* v.3.5.0 R package.

The pan-genome of each phylogroup and source was taken from the pan-genome of the species. The pan-genome of the MGE (called Pan-MGE) was also taken from the species pan-genome and contained only genes encoding for MGEs.

### Rarefaction of pan-genomes

The number of singletons was strongly correlated to the number of genomes analyzed in each phylogroup (Pearson’s correlation = 0.97, P<10^−4^), indicating that the pan-genomes size depend on the number of genomes analyzed. Thus, to compare genetic diversity across datasets (e.g. phylogroups), we rarefied the genome datasets, *i*.*e*., each pan-genome was constructed with the same number of genomes in each comparison. To do this, 1,000 subsets of X genomes (X depending on the analysis, specified in the results section) were randomly selected for comparison in each group, resulting to datasets called hereafter *rarefied* datasets (S9 Fig).

### Identification of persistent-genomes

Gene families that are persistent were taken from the analysis of pan-genomes. A gene family was considered as persistent when it was present in a single copy in at least 99% of the genomes. We found 2,486 persistent gene families when considering the 1,294 Australian genomes, representing 52% of the average genome.

### Identification of core-genome

The core genome was taken from the analysis of the pan-genome. A gene family was considered as core if it is present in one single copy in all the genomes. To assess the nucleotide diversity, we built a core-genome with all the genomes of the ECOR, RefSeq, and Australian datasets. It was composed of 112 core gene families. Each gene family was aligned with mafft v.7.222 (using FFT-NS-2 method) [110], and used to compute the average nucleotide diversity (π) in each dataset and within each phylogroup (see above).

### Functional assignment of the pan-genome

Gene functional assignment was performed by searching for protein similarity with hmmsearch from HMMer suite v.3.1b2 [111, 112] on the bactNOG subset of the EggNOG v.4.5.1 database [47]. We have kept hits with an e-value lower than 10^−5^, a minimum alignment coverage of 50% of the protein profile, and when the majority (>50%) of non-supervised orthologous groups (NOGs) attributed to a given gene family pertained to the same functional group (category). The gene families that cannot be classified into any existing EggNOG clusters were grouped into the “unknown” category. Hits corresponding to poorly characterized or unknown functional EggNOG clusters were grouped into the “poorly characterized” category.

### Phylogenetic analyses

We built a rooted phylogeny of the species in two steps. **The phylogenetic species tree of Australian *E*. *coli*** was reconstructed from the concatenated alignments of the 2,486 persistent genes of the 1,294 Australian *E*. *coli* strains (see S3 Fig for a description of the method). The alignment was done using the corresponding protein sequences with mafft v.7.222 (using FFT-NS-2 method) [110]. Protein alignments are more accurate and produce codon-based alignments that can be used for population genetics analysis. Since at this evolutionary distance the DNA sequences provide more phylogenetic signal than protein sequences, we back-translated the alignments to DNA, as is standard usage. This involved replacing every amino acid in the alignment by the original codon. Hence, the DNA sequence remains unchanged after translation and back-translation. We built phylogenies from persistent genomes to avoid the loss of signal associated with the small core genomes. When a genome lacked a member of a persistent gene family, or when it had more than one member, we added a stretch of gaps (‘-‘) of same length as the other genes for it in the multiple back-translated alignments. Adding a few "-" has little impact on phylogeny reconstruction. For example, Filipski et al [113] showed that adding up to 60% of missing data in the alignment matrix could be informative. In our study, only 0.3% of the genes are missing in the matrix and the effect of missing data should be negligible relative to the advantage of using the phylogenetic signal from 2,486 persistent genes instead of only the one of 295 core genes (S3C Fig). We have not removed recombination tracts from the multiple alignment because this has been shown to amplify errors in determining phylogenetic distances and it usually does not affect the topology of the tree [114, 115]. If determination of the recombination was accurate in our >1,300 genomes dataset, this would have led to the exclusion of almost all the genes. The length of the resulting alignment for the species was 2,298,168 bp. Each tree was computed with IQ-TREE multicore v.1.6.7 [116] under the GTR+F+I+G4 model. This model gave the lowest Bayesian Information Criterion (BIC) among all models available (option–m TEST in IQ-TREE). We made 1,000 ultra-fast bootstraps to evaluate node support (options–bb 1000 –wbtl in IQ-TREE) and to assess the robustness of the topology of each tree [117].

**The phylogenetic tree of *Escherichia* genus** was inferred from the persistent-genome obtained with the 87 outgroup genomes and the 100 *E*. *coli* reference genomes (see above) using the same procedure as the species tree. In this case, the persistent-genome is composed of 1,589 gene families, and the resulting alignment of 1,469,523 bp. The genus phylogenetic tree was extremely well supported: all nodes had bootstrap support higher than 95%. Its topology was consistent with a previous study [118] (S4C Fig). Then, we used it to precisely root the species tree (S4D Fig).

**The most recent common ancestor of each phylogroup**: We identified the node corresponding to the most recent common ancestor (MRCA) for each phylogroup from the rooted species tree using the *findMRCA* function from the *phytools* v.0.6.44 R package. Then, the subtree of each phylogroup was extracted using the *extract*.*clade* from the *ape* v.5.2 R package [119]. The distance to the MRCA was computed from the length of branches in each subtree. It corresponds to the average depth (distance from the MRCA) of all genomes (tips) within a phylogroup and was inferred using the *depthTips* from the *phylobase* v.0.8.6 R package (https://github.com/fmichonneau/phylobase).

### Evolutionary distances

For each pair of genomes, we computed a number of measures of similarity: 1) The **Patristic distance** was computed from the length of branches in the *Australian E*. *coli* species phylogenetic tree. The patristic distance is simply the sum of the lengths of the branches that link two genomes (tips) in the tree, and was inferred using the *cophenetic* function from the ape v.5.2 R package [119]. They were computed between all pairs of genomes, of the same ST (*intra-ST*), of different ST (*inter-ST*) within identical phylogroup, or of different phylogroups (*inter-phylogroup*). As expected, we found that the *intra-phylogroup (both intra-ST and inter-ST)* patristic distances were significantly shorter than the *inter-phylogroup* (Wilcoxon test, P<10^−4^). 2) **The Gene Repertoire Relatedness index** (GRR) between two genomes was defined as the number of common gene families (the intersection) divided by the number of genes in the smallest genome [120]. It is close to 100% if the gene repertoires are very similar (or one is a subset of the other) and lower otherwise. 3) **The Manhattan index** between two genomes is the number of different gene families. If two genomes have identical gene content, the corresponding Manhattan index is 0. 4) **The Jaccard index** between two genomes was defined as the number of common gene families (the intersection) divided by the number of gene families in both (the union). The Jaccard index between two genomes describes their degree of overlap with respect to gene family content. If the Jaccard distance is 1, the two genomes contain identical protein families. If it is 0 the two genomes are non-overlapping.

To characterize the genetic diversification of each phylogroup of the Australian dataset, we computed the three different standard indexes: the GRR, the Jaccard, and the Manhattan indexes. All these indexes were highly correlated (S10B Fig). Thus, only analyses with GRR were reported and illustrated in the main text. Note that we always used the matrix of presence/absence of gene families to compute all these indexes, meaning that multiple occurrences were not considered. This downplays the impact of IS on pan-genome size and makes more conservative estimates of GRR divergence.

### Reconstruction of the evolution of gene repertoires

We assessed the evolutionary dynamics of gene repertoires of the Australian genomes using Count (downloaded in January 2018) [121] with the Wagner parsimony method. Due to the size of our dataset it was not possible to do the analysis using birth-death models, but our previous analyses revealed very few differences between the two methods in smaller datasets [122]. Wagner parsimony penalizes the loss and gain of individual family members (with relative penalty of gain with respect to loss of 1, option g = 1), and infers the history with the minimum penalty. Thus, from the pan-genome, *i*.*e*., the matrix of presence-absence of gene families, and the rooted species tree, Count inferred the most parsimonious gain/loss scenario of each gene family along the tree. At each tree node, Count detailed information about individual families: presence/absence, and family events on the edge leading to the node. Hence, we have reconstructed the gene content of ancestral genome at each node. At each terminal branch, the expected total number of recent acquisitions (HGT) was computed by summing all family-specific gene gains obtained from the edge leading to the tip. Among them, we identified MGE associated genes that were recently acquired in each genome. We applied a similar strategy to identify recent losses.

### Distribution of accessory families across phylogroups (or sources)

We counted the number of MGE-associated gene families across phylogroups (Fig 5A) or sources (S15 Fig). We excluded the singletons from this analysis to avoid over-estimation of the number of families specific to one category. To test if some categories over-represented or under-represented these genes, we made 1,000 simulations. In each simulation, we shuffled the phylogroup (or source) assignment of the genomes while keeping the same number of taxa in each category (phylogroups or sources). Thus, the presence of a gene family in a genome and its frequency in the pan-genome remains the same, only the phylogroup (or the source) of genomes changes. The Z-score obtained for the observed number in the real data with respect to the random distribution (from 1,000 simulations) was reported for each case with a color code ranging from blue (under-representation, Z-score<-1.96) to red (over-representation, Z-score>1.96).

### Recent co-occurrence of gains (co-gains) of gene families within phylogroups

We counted the number of recently acquired gene pairs (co-gains) from the same pan-genome gene family (see above) within and between phylogroups. Recently acquired genes were defined as those inferred as acquired in terminal branches using Count. To test if some phylogroups over-represented or under-represented these co-gains, we compared the observed number (O) within each phylogroup to the expectation (E) given by 1,000 simulations. In each simulation, we shuffle the phylogroup assignment of the taxa (same approach as for the accessory gene families) and count the number of co-gains within and between phylogroups. For each phylogroup, we then divided the number observed in the real data (O) by the average number observed in the simulations (E), and computed the Z-score of the observed number (O) with respect to the random distribution (E). We considered an over(under)-representation significant when Z-score>1.96 (Z-score<-1.96). Note that the O and E numbers had to be previously normalized (divided by the total number of gene pairs, i.e. the sum of pairs within and between phylogroups, in the real data, and in each simulation, resp.). We applied the same approach (i) considering only gene pairs encoding for MGEs (similar result as in Fig 5), (ii) for sources (instead of phylogroups, Fig 6).

### Network of co-occurrence of gains (co-gains) of gene families across phylogroups

All co-gains (see above) were split into all possible combinations of phylogroup pairs (21 combinations). To test if these co-gains are over- or under-represented between phylogroups, we compared the observed number (O) between each phylogroup to the expectation (E) given by 1,000 simulations with the same strategy as above. As before, we normalized the observed and expected numbers by the total number of co-gains in each simulation, calculated the (O/E) ratio, and the Z-score of each observed value in the real data with respect to the random distribution (E). The network was drawn using the *igraph* v.1.2.2 R package (https://igraph.org/r/) with the circle layout option, where nodes are phylogroups, edges are (O/E) values for which the Z-score is significantly different from zero. The width of the edges is proportional to the (O/E) value and the color is blue for under- and red for over-representation (Fig 5C). We applied the same approach considering only gene pairs encoding for MGEs (S16 Fig).

### Gene family diversity

We computed Shannon indexes to assess the diversity of each gene family recently acquired (terminal branches) across phylogroups and across sources (Fig 6E). If diversity is low, this means that acquisitions are clustered by phylogroup or source (depending on the analysis). For this, we calculated the relative frequency of each gene family recently acquired within each phylogroup (vs. each source). It is simply the number of genomes (within a phylogroup) with at least one acquisition divided by the total number of genomes in the phylogroup. We therefore obtained 2 vectors per gene family (one for phylogroups and one for sources) each containing 7 frequencies (for each phylogroup or each source) and then applied for each vector the function *diversity* from the *vegan* v.2.4.6 R package (https://github.com/vegandevs/vegan). If the index is 0, recent acquisitions of genes of the family are limited to a single group (phylogroup or source). The higher the index, the more scattered the acquisitions of the family's genes are (across phylogroups or sources).

### GWAS

We studied the association between the pan-genome, i.e., the matrix of presence-absence of gene families, and different phenotypes (i.e., phylogroups, and sources) using Scoary v.1.6.16 [123]. The method used the rooted species tree to correct for phylogenetic dependency. To correct for multiple comparisons, only gene families with a Bonferroni-adjusted p-value < 10^−10^ were selected. In the case of phylogroups, more stringent thresholds were applied, i.e., p-value 10^−20^. We used the odds ratio (R) to determine whether the gene is positively (R>1) or negatively (R< = 1) associated with the tested phenotype. Analyses of the whole pan-genome or excluding all singletons produced similar results. A complete list of gene families positively and negatively associated with each phenotype is described in S2 Dataset. The sequence of one gene from each family is also available in the S2 Dataset, to facilitate the use of these results by the community.

### Statistics

All basic statistics were performed using R v 3.5.0, or *JMP*-13. (i) **Analysis of means**: We used **ANOM** to compare group means to the overall mean, when the data were approximately normally distributed. In cases where the data were clearly non-Gaussian and could not be transformed, we used the nonparametric version of the ANOM analysis, i.e., **ANOM with Transformed Ranks**. It compares each group’s mean transformed rank to the overall mean transformed rank. In both, we used the methods implemented in *JMP*-13. (ii) **Pairwise Wilcoxon Rank Sum Tests** were computed using the *pairwise*.*wilcox*.*test* function from the *stats* v.3.5.0 R package. We used the Bonferroni correction during multiple comparison testing. (iii) **Fisher's exact tests** were computed using the *fisher*.*test* function from *stats* v.3.5.0 R package. They were performed for testing the null of independence of rows (phylogroups) and columns (sources) in a 2x2 contingency table. (iv) **Correlation coefficients.** Pearson’s and Spearman’s rank correlation rho were computed using the *cor* function from *stats* v.3.5.0 R package. The correlation matrices were represented using the *corrplot* v.0.84 R package (https://cran.r-project.org/web/packages/corrplot/index.html). (v) **Smooth regression**: We used the generalized additive model (*gam)* smoothing method from the *mgcv* v.1.8.23 R package (https://cran.r-project.org/web/packages/mgcv/index.html). (vi) **Stepwise multiple regressions** were computed with *JMP*-13. This standard statistical method consists in a stepwise integration of the different variables in the regression by decreasing order of contribution to the explanation of the variance of the data [124]. We used the forward algorithm and the BIC criterion for model choice in the multiple stepwise regressions. The P-values associated with each variable were assessed using an F-test.

### Identification of Mobile Genetic Elements (MGEs)

**Prophages:** Prophages were predicted using VirSorter v.1.0.3 [53] with the RefSeqABVir database in all genomes from Australian and RefSeq datasets, as a control. The least confident predictions, *i*.*e*., categories 3 and 6, were excluded from the analyses in both datasets. The prophage-associated regions in drafts are more numerous and shorter than in complete RefSeq genomes (S11 Fig). These results reveal that such regions are sometimes split in assemblies. In complete genomes, the cumulative size of the prophage-associated regions (X) is highly correlated with the number of prophages (Y) present in the genomes (Y = 1.2923362 + 1.6767.10^−5^ X, R^2^ = 0.91, P<10^−4^, S11 Fig). Hence, we used this linear equation to estimate the number of prophages in drafts using the cumulated size of prophage regions in the draft genomes. **Plasmids**: In the RefSeq dataset, all the extrachromosomal replicons were considered as plasmids. In the Australian dataset, plasmid sequences were identified using PlaScope v.1.3 [54] with the database dedicated to *E*. *coli*. PlaScope provides a method for plasmid and chromosome classification of *E*. *coli* contigs. It has the specificity to select a unique assignment to each contig of a draft genome to plasmid, chromosome or unclassified. The number (~16, max: 124) and size (~9 kb, max: 166 kb) of contigs predicted as plasmid were highly variable (S12 Fig) in the Australian dataset. Their size is much smaller than that of the average plasmid in complete genomes (~80 kb), reflecting the split of plasmids across different contigs because of the presence of repeated sequences, *e*.*g*. IS elements. Hence, we have not attempted to estimate the exact number of plasmids per genome and focus our analysis on the number of genes predicted to be in plasmid contigs. **MGEs (Plasmids + Prophages)**: We found 11,864 gene families specifically related to plasmid elements, 14,188 to prophage elements, and 2,599 shared by both (9% of the MGEs gene families). In complete genomes, prophage and plasmids elements account for half of the pan-genome, of which 1 third were singletons. The large fraction of singletons from MGEs confirms that these elements are extremely diverse and evolved very rapidly, which underlines the difficulty of accurately detecting them and probably leads to their under-estimation in draft genomes. **Loci encoding conjugative or mobilizable elements** were detected with the CONJscan module of MacSyFinder [125], using protein profiles and definitions following a previous work [55, 126]. 87% of conjugative systems and 75% of putative mobilizable elements were located on contigs predicted as plasmids by Plascope. **Integrons** were identified using IntegronFinder v.1.5 with the–local_max option [58]. 186 integron-integrase (*intI*) were detected with one quarter located at the edges of contigs. We only found one copy per genome. They were often located on very short contigs (20 proteins on average), and five make all the contigs. Most (86%) were located on contigs predicted as plasmid by Plascope, the remaining were on unclassified contigs. Except for the latter, *intI* genes were always located next to ARGs. **IS elements** were identified using ISfinder [57]. Only hits with an e-value lower than 10^−10^, a minimum alignment coverage of 50% and with at least 70% identity were selected, we extracted the IS name of the best hit. Therefore, we identified 47,592 genes encoded for IS elements, among them 43% were located at the edges of contigs (20,329/47,592). They represented 1,006 gene families (~1% of the pan-genome), of which 41% were singletons. Only 13% were multigenic protein families (*i*.*e*., with more than one member in at least one genome). Among them, 9 protein families were found in more than 10 copies in at least one genome, i.e., ISEc1 (10 copies), IS1397 (11), ISSoEn2 (11), IS621 (11), IS2 (15), IS629 (17), IS200C (17) IS1203 (18), and the most extreme case IS1F (107). Very large numbers of ISs, usually a sign of recent proliferation, was restricted to a small number of genomes (S1 Dataset), but this may be an under-estimate caused by the loss of ISs in the assembling process. ISs were often fragmented, characterized by numerous singletons, and six times more frequently present at the edges of contigs than expected by chance. All the results are reported in S1 Dataset.

### Capsule systems

We used CapsuleFinder as published in [127] to search for Group I (Wzy-dependent), Group II and III (ABC-dependent), Group IV (subtypes e, f and s), synthase-dependent (subtypes cps3-like and hyaluronic acid) and PGA (Poly-γ-d-glutamate) capsules in the genome database. This allowed the detection of 2,829 systems: 1,236 Group I, 123 Group II, 777 Group IV e and 693 Group IV s. All the results are reported in S1 Dataset.

Antibiotic resistance genes (ARG) were detected using 2 curated databases of antibiotic resistance protein: Resfinder v.3.1 [128] and ARG-ANNOT v.3 [129]. Therefore, we used BlastP and selected the hits with an e-value lower than 10^−5^, with at least 90% of identity and a minimum alignment coverage of 50%. We found a strong positive correlation between the number of ARGs per genome using each database (pearson’s r = 0.97, P<10^−4^). The main difference is the additional detection of three ARGs by ARG-ANNOT, i.e., AmpC2, AmpH, Mfd, which are persistent in Australian dataset and normally do not confer antibiotic resistance in *E*. *coli*. All the results are reported in S1 Dataset.

Virulence factors (VF) were identified using VFDB (downloaded in February 2018, [65]). The two databases, *i*.*e*., VFDB_setA and VFDB_setB were used independently. We used BlastP and selected the hits with an e-value lower than 10^−5^, at least 70% of identity and minimum alignment coverage of 50%. We found 1,332 (vs. 3481) gene families encoding virulence factors with the setA (vs. setB). In spite of these differences, we found qualitatively similar conclusion with the 2 sets because they are very correlated (pearson’s r = 0.97, P<10^−4^). All the results are reported in S1 Dataset.

## Supporting information

S1 Text Isolates description. (DOCX)

S2 Text Quality control of the genomic sequences. (DOCX)

S3 Text Effect of contig breaks on the estimates of pan-genomes. (DOCX)

S1 Table Overall diversity of the three datasets. (PDF)

S2 Table Genetic diversification across phylogroups of Australian dataset. (PDF)

S3 Table The effects of phylogroup and the strains’ source on genome size: results of the stepwise multiple regression. Stepwise regression is an approach to selecting a subset of parameters (among the strains’ source and phylogroup) for a regression model. In forward selection, terms are entered into the model and most significant terms are added until all of the terms are significant. We used the minimum Bayesian Information Criterion to choose the best model. The Stepwise regression report (1) shows the statistics of the best model. As each step is taken, the Step History report (2) records the effect of adding a term to the model, and shows the order in which the terms entered the model and the statistics for each model. The Current Estimates report (3) indicates whether a term is currently in the best model and shows the statistics of each term for this model.
(PDF)

S4 Table The effects of phylogroup and the strains’ source on genome size without MGE: results of the stepwise multiple regression. Same approach described in S3 Table.
(PDF)

S5 Table Genetic diversification across sources of Australian dataset. (PDF)

S6 Table The effects of phylogroup and the strains’ source on MGE content: results of the stepwise multiple regression. Same approach described in S3 Table.
(PDF)

S1 Dataset The main characteristics of each genome of this study. (XLSX)

S2 Dataset Association of the pan-genome with phylogroups and isolation sources: results of the GWAS analyses. (XLSX)

S1 Fig General genomic characteristics of the 1,294 Australian *E*. *coli* genomes. A. Histogram and boxplot of genomic features, *i*.*e*., the genome size (Mb), the number (#) of genes encoding proteins, the GC content (GC%), the gene density, the number of essential genes, the number of contigs and the L90 (Methods). For each case, the dash line corresponds to the smoothed curve, the red arrow to the median and the blue arrow to the average of each distribution. B. Strong positive correlation between the genome size and the number of genes (spearman’s rho = 0.98, P<10^−4^). C. Weak positive correlation between the genome size and the number of contigs (spearman’s rho = 0.23, P<10^−4^). The genomes with the greatest number of contigs were not necessarily the largest. Linear regression (dash line) and statistics were reported.
(EPS)

S2 Fig The large Australian *E*. *coli* pan-genome. A. Number of gene families according to their occurrence in genomes. Singletons (in green), *i*.*e*., genes present in a single genome, represent 44% of the pan-genome. Persistent gene families (in gold), *i*.*e*., present in at least 99% of genomes, represent only 3% of the pan-genome. B. Fraction of gene families (%) according to their frequency among the pan-genome and the average genome. Frequencies were represented by a color code ranging from light grey (present in less than 1% of genomes) to black (up to 99%), persistent genes (>99%) were represented in gold. 82% of the gene families are rare, *i*.*e*., present in less than 1% of genomes including the 33,705 singletons. Persistent gene families represent 53% of the average genome, while singletons less than 1%. C. Rarefaction curve of the full pan-genome and of the pan-genome after removing the 33,705 singletons (wo. S). In each case, we used 1,000 permutations (genomes orderings) and then averaged the results. The *alpha* (inferred using the heaps’ law model) is lower than 1 in both, indicating that the pan-genome is open in both. D. Rarefaction curve of the persistent genome (in gold) and of the core genome (in red), *i*.*e*., the cumulative number of gene families shared by 100% of the genomes. The evolution of the average number of new genes per genome is also reported (in green). When considering 1,294 genomes, there is on average 2,486 persistent proteins and only 26 singletons per genome. E. Violin-plots of the average sequence identity [left, mean], and the minimal sequence identity [right, min] observed in each of the 2,486 persistent gene families. The observed average sequence identity is 98.3% across families of persistent genes. The average minimal value observed across persistent gene families is 95.5%.
(EPS)

S3 Fig Construction of the concatenated alignments of persistent gene families. A. Graphical representation of the different steps of the phylogenetic trees build process from the persistent genome. Among persistent gene families, there are families that are core (present in 100% of the genomes, in red) and the remaining that have missing genes (not-core, in gold). B. Number of persistent gene families according to their number of missing genes in the Australian dataset. Only 12% of families are core, i.e., present in all genomes (in red). C. Violin-plot of the number of missing genes per genome in the Australian dataset. On average, the number of missing genes is around 8 per genome. It can reach up 93 in a single genome, but this represents less than 4% of persistent families.
(EPS)

S4 Fig The genus and species phylogenetic trees. A. Distance tree of 1,294 Australian *E*. *coli* and 86 outgroups genomes performed from the matrix of mash distances computed between all pairs of genomes using bionj. The number of genomes in each species (or clade) was indicated. The different phylogroups of *E*. *coli* were displayed: A (in blue), B1 (in green), E (in purple), D (in yellow), F (in orange), G (in brown) and B2 (in red). B. Boxplot of the mash distances computed between all pairs of genomes belonging to the same species (or clades). In both cases, the maximal mash distance was lower than 0.05. For *E*. *coli* species, the median was around 0.027 and the maximal value was 0.04. C. Phylogenetic tree of 100 Australian *E*. *coli* genomes representative to the diversity of the dataset and 86 outgroups genomes performed from the persistent-genome of the genus with IQ-TREE under the GTR+F+I+G4 model. We made 1,000 ultra-fast boostrap to assess the robustness of the topology of the tree. We found that all boostrap supports were higher than 95%. D. We rooted the species phylogenetic tree from the genus phylogenetic tree. The resulting rooted species tree was reported, and for simplicity, the main phylogenetic groups were collapsed.
(EPS)

S5 Fig Singleton characterization. A. Boxplots of gene size (bp) in the three categories of gene families, *i*.*e*., persistent (in gold), accessory (in grey) and singleton (in green). The average was represented by a black dot. The pairwise Wilcoxon Rank Sum test with bonferroni correction was applied to all comparisons (P<0.001 :***). B. Same analysis as in A, but distinguishing the genomic location of the gene of each set : inside of contigs (I, dark color) or at the edge of contigs (E, light color). The average gene size for each case was reported in the table. C. Percentage of genes located inside contigs (dark color) or at the edge of contigs (light color) in the 3 sets. The last column corresponds to the fraction of the 3 sets located at the edge of contigs. D. Heatmap of the observed/expected (O/E) ratios of genes located inside or at the edges of contigs in the 3 sets. The ratio (O/E) was reported for all comparisons with a color code ranging from blue (under-representation) to red (over-representation). The level of significance of each Fisher’s exact test was also indicated (P<0.001 :***). It was performed on each 2*2 contingency table. E. Fraction of singletons with no hit (in light gey), with a small domain (in grey) or fully included (black) in larger accessory or persistent gene families (S3 Text). F. Violin plots of the number of singletons (in green) or persistent (in gold) observed in the rarefied Australian and RefSeq datasets. In each case, 1,000 permutations of 50 randomly selected genomes were performed (i.e., we used rarefied datasets). The boxplot is in white and the mean is represented by a black dot. While the average number of singletons is significantly higher (30% more) in the rarefied Australian dataset (Wilcoxon test, P<10^−4^), the average number of persistent is also significantly higher (5% more, P<10^−4^) than the rarefied RefSeq dataset. Singletons represent 43%, and 35% of the rarefied Australian and RefSeq pan-genomes, resp.
(EPS)

S6 Fig Association between GRR (%, Gene Repertoire Relatedness) and the patristic distance of each pair of genomes. Here, the GRR were computed excluding singletons in all genomes. Due to the large amount of comparisons (points), we divided the plot area in regular hexagons. Color intensity is proportional to the number of cases (count) in each hexagon. The linear fit (full line, linear model (lm)) and the spline fit (dash line, generalized additive model (gam)) were reported for the whole (in black, all the species) or the intra-ST (in blue) comparisons. There was a significant negative correlation between GRR and the patristic distance (spearman’s rho = -0.69, P<10^−4^). The summary of the linear fit was: Y = 90.722391–76.2919X, R^2^ = 0.50,P<10^−4^. Hence, with or without singletons, the results were similar.
(EPS)

S7 Fig Comparison of Australian, ECOR and RefSeq datasets. A. Violin plots of the nucleotide diversity per site in the 3 datasets computed from the multiple alignments of 112 core gene families (see Methods). The pairwise Wilcoxon Rank Sum test with bonferroni correction was applied to all comparisons (P>0.05:ns). B. Rarefaction curve of the full pan-genomes of the 3 datasets. In each case, we used 1,000 permutations (genomes orderings) and then averaged the results. C. Violin plots of the size of the pan-genomes computed from the three rarefied datasets: In each case, 1,000 permutations of 50 randomly selected genomes were performed to calculated the rarefied pan-genomes. The pairwise Wilcoxon Rank Sum test with bonferroni correction was applied to all comparisons (P<10^−3^ :***). D. Average number of persistent (in gold), accessory (in grey) and singleton (in green) in the rarefied pan-genomes of each dataset.
(EPS)

S8 Fig Intra- and Inter-phylogroup genetic diversity. A. Violin plots of the nucleotide diversity per site (left), the MASH (center) and the patristic distances (right) computed with/between genomes belonging to the same phylogroup (intra-phylogroup, in seagreen), to different phylogroups (inter-phylogroup, in purple), or all together (ALL, in darkgrey). In all cases, intra- and inter-phylogroup distributions were significantly different (Wilcoxon tests, P<10^−4^). B. Boxplots of the nucleotide diversity (left), the MASH (center) and the patristic distances (right) computed with/between genomes in each phylogroup. The pairwise Wilcoxon Rank Sum test with bonferroni correction was applied to all comparisons. Here, only the non-significant (ns : P> = 0.05) comparisons were indicated, all other were higly significant P<10^−4^. C. Density of the patristic distances between all pairs of genomes of the same phylogroup (*intra-phylogroup*). The dash vertical line corresponds to the median of each distribution. (A-B-C) In all cases, similar results were obtained with rarefied datasets (i.e., comparing 50 randomly selected genomes in each groups, thus ignoring the small G phylogroup).
(EPS)

S9 Fig Pan-genomes, Pan-MGE, and rarefied Pan-genomes of each phylogroup and isolation source. A. Size of the pan-genome in each phylogroup and in each isolation source. The pan-genome sizes were correlated to the number of genomes in each group, even after excluding the singletons from the analysis (both, adjusted R^2^>0.88, P<10^−4^). The Rarefaction curve of the pan-genomes of the full dataset was also reported (All, in black). B. Rarefaction curves of the pan-genomes of each phylogroup and of the full dataset (All). C. Rarefaction curves of the gene-families associated to MGE in each phylogroup and in the full dataset (All). D. Rarefaction curves of the pan-genomes of each isolation sources. In each case, (i) we used 1,000 permutations (genomes orderings) and then averaged the results (full line = mean, dash line = s.d), (ii) the pan-genomes remained open (with an *alpha* lower than one, see methods) that we considered them as a whole or without singletons, (iii) the boxplots of the rarefied pan-genomes (using a number of genomes = 50) were reported. The color code used was displayed in the insert (top right).
(EPS)

S10 Fig Gene repertoire relatedness (GRR) within and between phylogroups. A. Average GRR (%) computed between pairs of genomes belonging to the same phylogroup (intra-phylogroup) and to different phylogroups (inter-phylogroup). The color code used was displayed in the insert (top right). B. Correlation between the different distances and indexes, *i*.*e*., GRR, Manhattan, Jaccard, MASH and patristic, computed between pairs of genomes belonging to the same phylogroup (intra-phylogroup) with the whole dataset or excluding singletons (woS). Spearman’s rank correlation rho matrix. Positive correlations were displayed in red and negative correlations in blue color. Color intensity and the size of the circle were proportional to the correlation coefficients. The p-value of each correlation was highly significant (P<10^−4^). We found similar results with rarefied datasets, i.e., considering only 50 randomly selected genomes in each phylogroup. We also found higher correlation coefficients using all the comparisons (intra- and inter-phylogroup).
(EPS)

S11 Fig Detection and Estimation of the number of prophages. A. Boxplot of the number of regions detected as prophage-related by Virsorter in the 370 complete RefSeq GenBank genomes and in the 1,294 draft Australian genomes. These distributions were significantly different, on average the number of regions detected was significantly higher in draft than in complete genomes (Wilcoxon test, P<10^−4^). B. Boxplot and histogram of the size of the detected regions in complete and draft genomes. These distributions were significantly different (Wilcoxon test, P<10^−4^). On average the regions were almost 4 times larger in the complete genomes than in draft genomes and few regions (644) in draft genomes had a typical size of known dsDNA phages (around 44kb). (A-B) showed that prophage elements were less assembled and were probably divided into several small contigs. The large regions (>60 kb) in complete genomes corresponded to tandem elements (consecutive on the genomic sequence). Thus, the number of detected regions did not correspond to the number of prophages either in the complete genomes (due to tandem elements) or in the drafts genomes (the elements being fragmented). C. Strong association between the cumulative size of the detected regions (X) with the number of detected regions (Y). Linear regression (dash red line) and statistics were reported. D. Boxplot of the predicted number of prophage elements in both the complete and the draft genomes using the linear equation showed in (C) from the cumulative size of the regions detected by VirSorter. These distributions were significantly different (Wilcoxon test, P<10^−4^). On average, there was 6.0 prophages in complete genomes, and 4.25 in draft genomes. The medians of the two data sets were closer reflecting probably the assembly problem related to the presence of prophages in tandem combined with the fact that they are often genetically close (most of them are lambdoids). In each panel, the red arrow corresponds to the median and the blue arrow to the average of each distribution.
(EPS)

S12 Fig Detection of plasmid elements. A. Boxplot of the number of contigs classified as plasmid by PlaScope in the 370 complete RefSeq GenBank (Complete) genomes and in the 1,294 draft Australian genomes (Draft). All the extrachromosomal replicons of the complete genomes were perfectly identified as plasmid elements by PlaScope. Hence, results based on the extrachromosomal replicons or on the contigs detected as plasmid by PlaScope were identical (Complete*). The average number of contigs was eight times larger in draft genomes than in complete genomes (15.4 vs 1.9) and reached up to 124 contigs. B. Boxplot and histogram of the size of the contigs detected as plasmid in complete and draft genomes. These distributions were significantly different (Wilcoxon test, P<10^−4^). On average the contigs were almost 10 times larger in the complete genomes than in draft genomes (81 kb vs. 8.9 kb). We identified 2347, 562 and 53 contigs larger than 20, 50 and 100 kb, resp. (A-B) showed that plasmid elements were poorly assembled and probably divided into several small contigs. C. Boxplot of the fraction of the proteome encoding plasmid elements per genome (*i*.*e*., the cumulative number of proteins located on contigs classified as plasmid divided by the total number of proteins of the genome) in complete and draft genomes. These distributions were similar (Wilcoxon test, P>0.1) with an average of 3.2% in both.
(EPS)

S13 Fig General genomic characteristics of the mobilome of Australian *E*. *coli*. Three types of MGEs were detected, *i*.*e*., prophage (left column), plasmid (middle columns) and IS elements (right column). A. Histogram and boxplot of genomic features of each type of MGEs, *i*.*e*, the cumulative size of the elements per genome (Kb), the total number (#) of genes encoded by the elements per genome, the fraction of the genome encoding these elements per genome. For each case, the dash line corresponds to the smoothed curve, the red arrow to the median and the blue arrow to the average of each distribution. B. Histogram and boxplot of the number of conjugation systems per genome. C. Number of conjugative systems: (MPF) and isolated relaxases (MOB) detected in our dataset. The different MPF types were indicated and also their genomic location, *i*.*e*., located on a contig classified as plasmid or as chromosome by PlaScope.
(EPS)

S14 Fig Contribution of MGEs to genome size variation. A. Association between the genome size (*i*.*e*., # of genes per genome) and the total number of genes associated to the MGE elements. B. Histogram and boxplot of the genome size (in grey), and of the genome size without MGE (in red), i.e., after removing all the genes encoding MGE elements (in red). These distributions were significantly different (Wilcoxon test, P<10^−4^). C. Same representation as in (a), but distinguishing the different types of MGEs, i.e., prophage, plasmid and IS elements. (A-C) We found a strong correlation in each case. Linear regression (dash red line) and statistics were reported. Similar results were obtained with the genome size (Mb). D. Number of singletons (in green) and accessory gene families encoding MGEs. The fraction of the pan-genome encoding such elements was reported in each case (%).
(EPS)

S15 Fig Distribution of gene families related to MGEs across phylogroups and sources. Number of accessory gene families associated to prophage and plasmid present in one (i.e., phylogroup-specific) to seven phylogroups (A), or in one (i.e., source specific) to seven sources (B). The Z-score obtained for the observed number with respect to the expected distribution (as in Fig 5A, we randomized 1,000 times, only the phylogroup (A) or the source (B) assignment of genomes) was reported for each case with a color code ranging from blue (under-representation) to red (over-representation). The frequency of these families (average number of genomes) was also indicated in (C) for phylogroups, and in (D) for sources.
(EPS)

S16 Fig Network of recent co-occurence of gains (co-gains) of MGE genes within and between phylogroups. Nodes are phylogroups and edges the O/E ratio of the number of pairs of MGE genes (from the same gene family) acquired in the terminal branches of the tree. Only significant O/E values (and edges) are plottted (|Z-score|>1.96). Under-represented values are in dash blue and over-represented in red (see Methods).
(EPS)

S17 Fig Genome size and MGE content according to sources within each phylogroup. A. Heatmap of the average genome size of strains from different sources in each phylogroup. The deviation to the overall intra-phylogroup mean (i.e., the average genome size of all strains belonging to a given phylogroup) was reported for all comparisons with a color code ranging from blue (below average) to red (above average). The level of significance of each ANOM test was indicated (P> = 0.05 : ns; P<0.05 : *; P<0.01 : **; P<0.001 :***). It was performed within each phylogroup (each line). (B-C-D) Same representation as in (A), but in relation with the average number of genes associated to MGEs (B), to prophage (C), or plasmid elements (D).
(EPS)

S18 Fig Association of integrons and ARGs with human (or domesticated animals). A. Violin plots of the number of ARGs in genomes encoding integron-integrase (*int1+*) or not (*int1-*). The level of significance of the Wilcoxon test was indicated (P<10^−3^). B. Heatmap of the proportion of genomes *int1+* in each phylogroup and source. A cross marks the absence of data. C. Same as in (B) but we merged sources related to human activity (with), or not directly associated to human (without). The level of significance of each ANOM for proportions test was indicated (P> = 0.05 : ns; P<0.05 : *; P<0.01 : **; P<0.001 :***). Here, we compared response proportions for the X levels to the overall response proportion from the contingency table. This method uses the normal approximation to the binomial. Therefore, in some cases sample sizes were too small to be tested. D. Heatmap of the average number of ARGs per genome in each phylogroup and source. E. Heatmap of the average number of ARGs when we merged sources related (with) or not (without) to human activity. The level of significance of each non-parametric ANOM test (ANOM with Transformed Ranks) was indicated (P> = 0.05 : ns; P<0.05 : *; P<0.01 : **; P<0.001 :***). The deviation to the overall mean (i.e., in all genomes) was reported for all comparisons with a color code ranging from blue (below average) to red (above average). The color code used was displayed in the top of each panel.
(EPS)

S19 Fig Distribution of VFs and Colicins MGEs across phylogroups and sources. (A-B). Heatmap of the average number of VFs per strain from different sources in each phylogroup. The deviation to the overall mean (i.e., whole dataset, in A) or to the intra-phylogroup mean (i.e., the average number of all strains belonging to a given phylogroup, in B) was reported for all comparisons with a color code ranging from blue (below average) to red (above average). The level of significance of each ANOM test was indicated (P> = 0.05 : ns; P<0.05 : *; P<0.01 : **; P<0.001 :***). It was performed within each phylogroup (each line, in B). C. Heatmap of the average number of Colicins per genome in each phylogroup and source. D. Same representation as in (B), but in relation with the average number of Colicins per genome.
(EPS)

S20 Fig Distribution of capsule systems across phylogroups and sources. A. Heatmap of the average number of capsule systems per genome in each phylogroup and source. B. The deviation to the intra-phylogroup mean (i.e., the average number of all strains belonging to a given phylogroup) was reported for all comparisons with a color code ranging from blue (below average) to red (above average). The level of significance of each ANOM test was indicated (P> = 0.05 : ns; P<0.05 : *; P<0.01 : **; P<0.001 :***). It was performed within each phylogroup (each line). C. Prevalence (%) of each capsule groups across phylogroups and sources.
(EPS)
