# Supplementary material for: Phylogenetic background and habitat drive the genetic diversification of Escherichia coli
Source: PLoS Genet. 2020 Jun 12;16(6):e1008866. doi: 10.1371/journal.pgen.1008866 (PMC7314097; doi:10.1371/journal.pgen.1008866)
Supplement: S1 Text — (DOCX) [file pgen.1008866.s001.docx]

### **S1 Text: Isolates description**

The collections of strains from more than 3,300 non-human vertebrate hosts were acquired by sampling a single putative *E. coli* isolate from the feces of each host. They represent diverse species collected from across Australia [1-3]. The mammals sampled were assumed to be healthy hosts. The birds sampled represented a mix of presumably healthy birds collected from the wild, and native birds arriving at veterinarian clinics and wildlife rehabilitation centers. The phylogroup membership of these isolates was determined using the Clermont method[4]. The subset of strains selected for whole genome sequencing (WGS) was chosen to represent the diversity present in each phylogroup.

*E. coli* isolates were recovered from 306 samples of poultry meat purchased from retail outlets in the Australian Capital Territory [5]. Multiple isolates were collected per meat sample, characterized using REP-PCR, and assigned to phylogroups using the Clermont method. One example of each REP-type was selected for WGS.

Two collections of *E. coli* from more than 1,000 humans living in the Canberra region were aquired in 2002 and 2015 by taking a single isolate from unique urine, faecal or blood samples processed by the Canberra Hospital Microbiology Laboratory [6, 7]. A subset of strains were selected to represent the diversity present in each phylogroup. Another collection of *E. coli* from 69 humans living in the Canberra region was created by sampling up to 20 *E. coli* isolates from biopsies taken from up to five locations in the lower gut [8]. The isolates were characterised using REP-PCR and assigned to phylogroups using the Clermont method. One example of each REP-type present in a host was selected for WGS.

A collection of *E. coli* isolated from over 800 water samples collected from the Gold Coast region of Queensland, the Sydney region of New South Wales, and the Australian Capital Territory was created by taking up to 20 isolates from each sample [9]. The isolates were characterised using REP-PCR and assigned to phylgroups. For WGS, a subset of isolates was chosen to represent the diversity present in each phylogroup.

1. Gordon DM, FitzGibbon F. The distribution of enteric bacteria from Australian mammals: host and geographical effects. Microbiology. 1999;145 ( Pt 10):2663-71. doi: 10.1099/00221287-145-10-2663. PubMed PMID: 10537188.

2. Gordon DM, Cowling A. The distribution and genetic structure of Escherichia coli in Australian vertebrates: host and geographic effects. Microbiology. 2003;149(Pt 12):3575-86. doi: 10.1099/mic.0.26486-0. PubMed PMID: 14663089.

3. Blyton MD, Pi H, Vangchhia B, Abraham S, Trott DJ, Johnson JR, et al. Genetic Structure and Antimicrobial Resistance of Escherichia coli and Cryptic Clades in Birds with Diverse Human Associations. Appl Environ Microbiol. 2015;81(15):5123-33. doi: 10.1128/AEM.00861-15. PubMed PMID: 26002899; PubMed Central PMCID: PMCPMC4495204.

4. Clermont O, Christenson JK, Denamur E, Gordon DM. The Clermont Escherichia coli phylo-typing method revisited: improvement of specificity and detection of new phylo-groups. Environ Microbiol Rep. 2013;5(1):58-65. doi: 10.1111/1758-2229.12019. PubMed PMID: 23757131.

5. Vangchhia B, Blyton MDJ, Collignon P, Kennedy K, Gordon DM. Factors affecting the presence, genetic diversity and antimicrobial sensitivity of Escherichia coli in poultry meat samples collected from Canberra, Australia. Environ Microbiol. 2018;20(4):1350-61. doi: 10.1111/1462-2920.14030. PubMed PMID: 29266683.

6. Gordon DM, Stern SE, Collignon PJ. Influence of the age and sex of human hosts on the distribution of Escherichia coli ECOR groups and virulence traits. Microbiology. 2005;151(Pt 1):15-23. doi: 10.1099/mic.0.27425-0. PubMed PMID: 15632421.

7. Gordon DM, Geyik S, Clermont O, O'Brien CL, Huang S, Abayasekara C, et al. Fine-Scale Structure Analysis Shows Epidemic Patterns of Clonal Complex 95, a Cosmopolitan Escherichia coli Lineage Responsible for Extraintestinal Infection. mSphere. 2017;2(3). doi: 10.1128/mSphere.00168-17. PubMed PMID: 28593194; PubMed Central PMCID: PMCPMC5451516.

8. Gordon DM, O'Brien CL, Pavli P. Escherichia coli diversity in the lower intestinal tract of humans. Environ Microbiol Rep. 2015;7(4):642-8. doi: 10.1111/1758-2229.12300. PubMed PMID: 26034010.

9. Power ML, Littlefield-Wyer J, Gordon DM, Veal DA, Slade MB. Phenotypic and genotypic characterization of encapsulated Escherichia coli isolated from blooms in two Australian lakes. Environ Microbiol. 2005;7(5):631-40. doi: 10.1111/j.1462-2920.2005.00729.x. PubMed PMID: 15819845.
