# Supplementary material for: Phylogenetic background and habitat drive the genetic diversification of Escherichia coli
Source: PLoS Genet. 2020 Jun 12;16(6):e1008866. doi: 10.1371/journal.pgen.1008866 (PMC7314097; doi:10.1371/journal.pgen.1008866)
Supplement: S2 Text — (DOCX) [file pgen.1008866.s002.docx]

### **S2 Text: Quality control of the genomic sequences**

To assess the completeness of the draft genomes of the Australian and Outgroup datasets, we (i) computed the minimum number of contigs necessary to cover 90% of the genome (L90 value), (ii) the total number of contigs of each genome, and (iii) checked for the presence of known *E. coli* essential genes. ***L90 value***. We computed the L90 value for all draft genomes. A high L90 value means that the genome is scattered into many contigs. Thus, may be caused by abundant repeats, such as insertion sequences (ISs), or poor sequence quality. The distribution of L90 values showed a clear gap between 316 and 385. Therefore, we decided to put the limit at L90=316, and found six genomes with a higher value from this study. ***Number of contigs*.** We also computed the number of contigs for all draft genomes. The resulting distribution showed a clear gap between 796 and 947 contigs. Therefore, we decided to put the limit at 796 contigs, and found five genomes with a higher value. **E. coli *essential genes*.** We retrieved 296 essential *E. coli* K12-MG1655 genes from the database DEG 10 [1] (S1 Dataset). Among them, we excluded 14 genes coding for poison-antidote systems like TA and RMS, or related to phage elements, because they were expected not to be present in all genomes. Homologous genes (>80% identity) were identified in all *Escherichia* genomes (from Australian, RefSeq and Outgroup datasets) using usearch v.11 (option –usearch_global; id=0.8) [11]. The 282 remaining essential genes were all persistent (present in at least 99% of genomes) among the RefSeq dataset. However, some complete genomes were missing up to 6 essential genes. We found that 77% of draft genomes contained all essential genes, 17% were missing one gene, and only four genomes more than 6 essential genes. The latter were removed from further analysis. The results of these 3 independent tests were reported in S1 Dataset. In summary, 10 Australian *E. coli* draft genomes and one outgroup genome failed at least one of these tests and were removed from further analysis, leading to a final dataset of 1,294 Australian *E. coli* genomes and 87 outgroup genomes.

1. Luo H, Lin Y, Gao F, Zhang CT, Zhang R. DEG 10, an update of the database of essential genes that includes both protein-coding genes and noncoding genomic elements. Nucleic Acids Res. 2014;42(Database issue):D574-80. doi: 10.1093/nar/gkt1131. PubMed PMID: 24243843; PubMed Central PMCID: PMCPMC3965060.
