# Supplementary material for: Phylogenetic background and habitat drive the genetic diversification of Escherichia coli
Source: PLoS Genet. 2020 Jun 12;16(6):e1008866. doi: 10.1371/journal.pgen.1008866 (PMC7314097; doi:10.1371/journal.pgen.1008866)
Supplement: S3 Text — (DOCX) [file pgen.1008866.s003.docx]

### **S3 Text: Effect of contig breaks on the estimates of pan-genomes**

The genomes are not completely assembled, which may lead to the observation of spurious gene fragments and gene calling errors. To test the impact of this on the observed genetic diversity, we carried out four tests (S5 Fig). First, we compared the average gene size of the three family categories, and found that singletons were almost half the size of persistent (and accessory) genes (S5A Fig). The observation that many singletons are small suggests that they are partial genes. Second, to test if singletons were all at contig borders, we computed the fraction of each pan-genome category located at the edges of the contigs (S5C Fig). As expected, singletons were largely over-represented (6 times more, S5D Fig) at these positions (and those in this location were even smaller, S5B Fig). Nevertheless, only 3% of the edges of contigs correspond to singletons, showing that contig breaks only rarely lead to singletons. The low correlation observed between the number of singletons and the number of contigs supports this result (spearman’s rho= 0.27, P<10^-4^). Third, we searched for sequence similarities between singletons and the other two categories using Blast+ v.2.6.0, and found that 39% of them showed extensive sequence similarity (at least 80% of identity) to larger proteins from the accessory or persistent categories (S5E Fig). Finally, we tested if our genomes produced very different results when compared with completely assembled genomes (RefSeq) (S5F Fig). The comparison of the number of singletons and persistent genes in the rarefied Australian and RefSeq datasets (see Methods), showed that the number of persitent gene families was higher in our dataset. Singletons were 30% more numerous in the Australian genomes, but still represented one third (35%) of the pan-genome of RefSeq genomes. In summary, there is probably an over-prediction of singletons in the Australian dataset (probably one third of the singletons are parts of CDS), but this has a small impact on the definitions of accessory and persistent gene datasets. Together, these two categories make 42,000 different gene families, which remains an impressive number for a single bacterial species. The average number of singletons per genome in each phylogroup of the Australian dataset were not significantly different (Wilcoxon tests, P>0.05). Hence, this over-prediction is unlikely to affect most of our results.
