# Supplementary material for: Phylogenetic background and habitat drive the genetic diversification of Escherichia coli
Source: PLoS Genet. 2020 Jun 12;16(6):e1008866. doi: 10.1371/journal.pgen.1008866 (PMC7314097; doi:10.1371/journal.pgen.1008866)
Supplement: S1 Table — (PDF) [file pgen.1008866.s004.pdf]

		Datasets		
		Australian	RefSeq	ECOR
Number of genomes	#	1294	370	72
Min-Max Genome Size (GS)	Mb	4.20-6.02	3.98-6.02	4.50-5.59
ΔGS	Mb	1.82	2.04	1.09
Mean GS	Mb (Std Dev)	5.02 (0.27)	5.15 (0.32)	4.97 (0.26)
GC%	% (Std Dev)	50.64 (0.14)	50.66 (0.15)	50.62 (0.14)
Gene density %	% (Std Dev)	87.24 (0.79)	86.40 (1.36)	87.55 (0.57)
Sequence Type ST	Richness (NA*)	442 (38)	135 (16)	45 (1)
	α diversity**	7.53	6.10	5.08
H-type	Richness (NA*)	46 (15)	37 (10)	26
	α diversity**	4.73	4.37	4.27
O-group	Richness (NA*)	142 (563)	96 (24)	42 (6)
	α diversity**	5.91	5.50	5.03
O :H serotype	Richness (NA*)	311 (568)	175 (32)	58 (6)
	α diversity**	7.53	6.41	5.55
Nucleotide diversity Pi	Mean *** (Std Dev)	0.01078 (0.0078)	0.01157 (0.0084)	0.01089 (0.0081)
Average-genome	# of proteins	4683	4965	4635
Pan-genome	# of protein families****	75890	33041	18584
Persistent-genome (99%)		2486	2157	2494
Core-genome (100%)		295	667	2494

* number of untypable genomes (NA)
** Shannon index
*** average of nucleotide diversity (π) of 112 core gene families in each dataset (ECOR, RefSeq, Australian)
**** from matrix of presence/absence of gene families : gene amplifications were not taken into account
