# Supplementary material for: Phylogenetic background and habitat drive the genetic diversification of Escherichia coli
Source: PLoS Genet. 2020 Jun 12;16(6):e1008866. doi: 10.1371/journal.pgen.1008866 (PMC7314097; doi:10.1371/journal.pgen.1008866)
Supplement: S2 Table — (PDF) [file pgen.1008866.s005.pdf]

Australian dataset

		A	B1 (+C)	E	D	F	G	B2	ALL
Genome	#	312	291 (+18)	61	185	71	33	323	1294
Min-Max GS	Mb	4.42-5.81	4.20-5.52	4.80-5.74	4.74-6.02	4.77-6.00	4.62-5.31	4.51-5.51	4.20-6.02
Δ GS	Mb	1.39	1.32	0.95	1.27	1.24	0.69	1.00	1.82
Mean GS (1)	Mb (Std Dev)	4.92 (0.32)	4.89 (0.19)	5.22 (0.23)	5.15 (0.27)	5.22 (0.26)	5.07 (0.19)	5.06 (0.19)	5.02 (0.27)
Proteome (1)	Mean # of genes (Std Dev)	4611 (347)	4585 (208)	4811 (260)	4770 (317)	4843 (276)	4737 (203)	4730 (218)	4683 (286)
Proteome woMGE (1)		4259 (211)	4237 (122)	4448 (188)	4379 (179)	4450 (166)	4328 (105)	4325 (112)	4309 (174)
MGE (1)		352 (179)	349 (134)	364 (126)	391 (177)	393 (142)	409 (120)	405 (151)	375 (158)
Sequence Type ST	Richness (NA*)	92 (13)	145 (11)	29	57 (7)	20(2)	3	96 (5)	442 (38)
	α diversity(**)	4.97	6.55	3.74	5.01	3.41	0.87	4.81	7.53
H-type	Richness (NA*)	31 (8)	28	19	23 (1)	11	4	16 (6)	46 (15)
	α diversity(**)	3.98	3.94	3.53	3.61	2.58	1.16	2.93	4.73
O-group	Richness (NA*)	53 (166)	65 (131)	23 (26)	26 (90)	10 (37)	6 (20)	37 (93)	142 (563)
	α diversity(**)	4.99	5.17	4.14	3.48	2.67	2.41	4.05	5.91
O:H serotype	Richness (NA*)	143 (169)	178 (131)	35 (26)	95 (90)	34 (37)	13 (20)	228 (95)	311 (568)
	α diversity(**)	5.70	6.35	4.19	4.52	3.67	2.93	5.06	7.53
Nucleotide diversity Pi(1)	Mean ****	0.00425	0.00390	0.00616	0.00749	0.00680	0.00170	0.00451	0.01078
	Std Dev	0.00401	0.00449	0.00525	0.00547	0.00629	0.00251	0.00366	(0.0078)
Average-genome (1)	# of gene families***	4510	4505	4701	4669	4745	4619	4642	4589
Pan-genome		36712	32964	15097	24724	14536	9435	27766	75890
α heaps law		0.43	0.48	0.43	0.57	0.66	0.68	0.48	0.46
Persistent-genome		2475	2833	2714	2840	2636	3437	3069	2486
Pan/Average		8	7	3	5	3	2	6	17
Persistent/Average %		55	63	58	61	56	74	66	54
MGE		14011	13971	4759	9552	4868	2755	10161	28651
MGE/Pan %		38	42	32	39	33	29	37	38
Phage-related (1)	Mean # of genes (Std Dev)	199 (94)	223 (91)	222 (69)	247 (110)	228 (89)	231 (70)	250 (107)	228 (99)
Plasmid-related (1)		166 (135)	133 (97)	143 (92)	164 (133)	194 (103)	191 (101)	154 (94)	157 (113)
IS-related (1)		46 (21)	29 (15)	61 (39)	36 (22)	41 (20)	44 (18)	34 (14)	37 (19)
VFA (2)		95 (15)	102 (11)	115 (8)	116 (10)	113 (9)	113 (10)	124 (11)	109 (16)
VFB (2)		199 (29)	225 (21)	245 (15)	242 (17)	237 (16)	219 (12)	242 (25)	227 (29)
ARG Resfinder (2)		2.3 (2.2)	1.9 (1.8)	2.1 (1.3)	1.8 (1.9)	4.8 (3.8)	3.1 (2.6)	2.2 (2.0)	2.2 (1.2)
ARG Argannot (2)		5.4 (2.5)	5.0 (2.0)	5.3 (1.5)	4.5 (2.2)	7.9 (3.9)	6.2 (2.8)	4.2 (2.1)	5.0 (2.5)
int1+ (3)	% of genomes	16%	10%	8%	6%	39%	30%	16%	14%
CONJ+(2)		39%	54%	72%	53%	45%	58%	66%	54%
Rarefied-PanGenome (1,4) N=50	Mean # of gene families (Std Dev)	15935 (619)	14446 (525)	13890 (322)	14861 (486)	12784 (373)	NA (4)	13246 (551)	
Rarefied-Persistent (1,4) N=50		2367 (80)	2699 (132)	2831 (41)	2915 (77)	2838 (41)	NA (4)	2954 (77)	
Rarefied-MGE (1,4) N=50		6477 (335)	6369 (314)	4338 (140)	5921 (248)	4213 (161)	NA (4)	5046 (306)	

* number of untypable genomes (NA)

** Shannon index

*** matrix of presence/absence of gene families : gene amplifications were not taken into account

**** mean of 112 core genes (the same as those of the 3 datasets : ECOR, RefSeq and Australian)

(1) standard ANOM test

(2) non-parametric ANOM test

(3) ANOM for proportions

(4) rarefied datasets were computed from 1000 combinations of 50 distinct genomes, while G group contains only 33 genomes.
