# Supplementary material for: Phylogenetic background and habitat drive the genetic diversification of Escherichia coli
Source: PLoS Genet. 2020 Jun 12;16(6):e1008866. doi: 10.1371/journal.pgen.1008866 (PMC7314097; doi:10.1371/journal.pgen.1008866)
Supplement: S3 Table — Stepwise regression is an approach to selecting a subset of parameters (among the strains’ source and phylogroup) for a regression model. In forward selection, terms are entered into the model and most significant terms are added until all of the terms are significant. We used the minimum Bayesian Information Criterion to choose the best model. The Stepwise regression report (1) shows the statistics of the best model. As each step is taken, the Step History report (2) records the effect of adding a term to the model, and shows the order in which the terms entered the model and the statistics for each model. The Current Estimates report (3) indicates whether a term is currently in the best model and shows the statistics of each term for this model. (PDF) [file pgen.1008866.s006.pdf]

1- STEPWISE REGRESSION REPORT

Y	SSE (1)	DFE (2)	RMSE (3)	RSquare (4)	Rsquare Adj (5)	Cp (6)	p (7)	AICc (8)	BIC (9)
**Genome_size**	**5.9028E+13**	**1269**	**215674.8678**	**0.360**	**0.357**	**20.7448**	**6**	**34944.26**	**34980.23**

(1) SSE : Sum of squared errors for the current model

(2) DFE : Error degrees of freedom for the current model

(3) RMSE : Root mean square error (residual) for the current model

(4) RSquare : Proportion of the variation in the response that can be attributed to terms in the model rather than to random error.

(5) RSquare Adj: Adjusts R2 to make it more comparable over models with different numbers of parameters by using the degrees of freedom in its computation.
The adjusted R2 is useful in stepwise procedure because you are looking at many different models and want to adjust for the number of terms in the model.

(6) Cp : Mallow's Cp criterion for selecting a model.

(7) p : Number of parameters in the model, including the intercept.

(8) AICc : Corrected Akaike's Information Criterion

(9) BIC : Bayesian Information Criterion

2-STEP HISTORY REPORT

Y	Step		Parameter (12)	ACTION (10)	"Sig Prob" (11)	RSquare (4)	Cp (6)	p (7)	AICc (8)	BIC (9)
**Genome size (Mb)**	**1**	Source	**Source{Water&BF&HI&MF&HF-HE&PM}**	**Entered**	**1.04698E-54**	**0.174**	**386.276**	**2**	**35261.82**	**35277.25**
**Genome size (Mb)**	**2**	Phylogroup	**Phylogroup{B1&A-B2&G&D&F&E}**	**Entered**	**3.09628E-50**	**0.306**	**122.628**	**3**	**35041.09**	**35061.66**
**Genome size (Mb)**	**3**	Phylogroup	**Phylogroup{B2&G-D&F&E}**	**Entered**	**5.57312E-12**	**0.332**	**73.587**	**4**	**34995.46**	**35021.17**
**Genome size (Mb)**	**4**	Source	**Source{Water&BF-HI&MF&HF}**	**Entered**	**4.17722E-08**	**0.347**	**44.211**	**5**	**34967.29**	**34998.12**
**Genome size (Mb)**	**5**	Source	**Source{HE-PM}**	**Entered**	**5.98408E-07**	**0.360**	**20.745**	**6**	**34944.26**	**34980.23**
Genome size (Mb)	6	Source	Source{HI-MF&HF}	Entered	0.021480237	0.363	17.401	7	34940.97	34982.06
Genome size (Mb)	7	Phylogroup	Phylogroup{B2-G}	Entered	0.062954831	0.364	15.915	8	34939.52	34985.73
Genome size (Mb)	8	Source	Source{MF-HF}	Entered	0.050472158	0.366	14.067	9	34937.69	34989.03
Genome size (Mb)	9	Phylogroup	Phylogroup{B1-A}	Entered	0.04915779	0.368	12.183	10	34935.83	34992.28
Genome size (Mb)	10	Source	Source{Water-BF}	Entered	0.080586062	0.370	11.125	11	34934.78	34996.35
Genome size (Mb)	11	Phylogroup	Phylogroup{D-F&E}	Entered	0.165659919	0.371	11.202	12	34934.88	35001.56
Genome size (Mb)	12	Phylogroup	Phylogroup{F-E}	Entered	0.653360514	0.371	13.000	13	34936.73	35008.50
**Genome size (Mb)**	**13**		**Best model**	**Specific**		**0.360**	**20.745**	**6**	**34944.26**	**34980.23**

(10) ACTION : Entered = Indicates whether a term is currently in the model.

(11) "Sig Prob" : The significance level associated with the Wald/Score ChiSq test statistic based on nDF degrees of freedom. The "Sig Prob" is used to determine the next term to be included in the model.

(12) Parameter : Water=freshwater; BF=Bird Faecal; HI=Human Intestinal, MF = Mammal Faecal; HF = Human Faecal ; HE = Human Extra-intestinal; PM = Poultry Meat

3-CURRENT ESTIMATES REPORT

Y	ACTION (10)		Parameter (12)	Estimate (13)	nDF (14)	SS (15)	"F Ratio" (16)	"Prob>F" (17)	RSquare (4)	%Explained_ Variance
Genome_size	Entered		Intercept	5047531.05	1	0	0	1		
**Genome_size**	**Entered**	**Source**	**Source{Water&BF&HI&MF&HF-HE&PM}**	**-100800.31**	**3**	**1.7235E+13**	**123.508**	**3.4692E-70**	**0.174**	**48.2**
**Genome_size**	**Entered**	**Source**	**Source{Water&BF-HI&MF&HF}**	**-39909.963**	**1**	**1.3263E+12**	**28.513**	**1.1017E-07**	**0.016**	**4.3**
Genome_size		Source	Source{Water-BF}	0	1	1.2092E+11	2.603	0.10691475		
Genome_size		Source	Source{HI-MF&HF}	0	1	2.4573E+11	5.301	0.02148024		
Genome_size		Source	Source{MF-HF}	0	1	1.5761E+11	3.395	0.06563984		
**Genome_size**	**Entered**	**Source**	**Source{HE-PM}**	**-62298.379**	**1**	**1.171E+12**	**25.174**	**5.9841E-07**	**0.013**	**3.5**
**Genome_size**	**Entered**	**Phylogroup**	**Phylogroup{B1&A-B2&G&D&F&E}**	**-97045.169**	**2**	**1.3348E+13**	**143.482**	**6.6454E-57**	**0.132**	**36.8**
Genome_size		Phylogroup	Phylogroup{B1-A}	0	1	1.0314E+11	2.219	0.13653071		
**Genome_size**	**Entered**	**Phylogroup**	**Phylogroup{B2&G-D&F&E}**	**-54784.388**	**1**	**1.8827E+12**	**40.475**	**2.7748E-10**	**0.025**	**7.1**
Genome_size		Phylogroup	Phylogroup{B2-G}	0	1	1.3688E+11	2.947	0.08627447		
Genome_size		Phylogroup	Phylogroup{D-F&E}	0	1	1.523E+11	3.280	0.07035921		
Genome_size		Phylogroup	Phylogroup{F-E}	0	1	1.7961E+10	0.386	0.53455457		

(13) Estimate :The current parameter estimate, which is zero if the effect is not currently in the model

(14) nDF : The number of degrees of freedom for a term. A term has more than one degree of freedom if its entry into a model also forces other terms into the model.

(15) SS : The reduction in the error (residual) sum of squares (SS) if the term is entered into the model or the increase in the error SS if the term is removed from the model.

(16) F ratio: The traditional test statistic to test that the term effect is zero. It is the square of a t-ratio.

(17) Prob>F : The significance level associated with the F statistic.
