# Supplementary material for: Phylogenetic background and habitat drive the genetic diversification of Escherichia coli
Source: PLoS Genet. 2020 Jun 12;16(6):e1008866. doi: 10.1371/journal.pgen.1008866 (PMC7314097; doi:10.1371/journal.pgen.1008866)
Supplement: S5 Table — (PDF) [file pgen.1008866.s008.pdf]

Australian dataset

		BF	PM	HE	HI	HF	MF	Water	ALL
Genome	#	195	283	112	172	93	135	285	1294
Min-Max GS	Mb	4.45-6.02	4.6-6.00	4.62-5.46	4.63-5.52	4.54-5.55	4.42-5.74	4.20-5.64	4.20-6.02
Δ GS	Mb	1.57	1.44	0.84	0.89	1.01	1.32	1.44	1.82
Mean GS (1)	Mb (Std Dev)	4.93 (0.24)	5.22 (0.24)	5.10 (0.18)	4.99 (0.19)	5.02 (0.22)	5.01 (0.22)	4.87 (0.24)	5.02 (0.27)
Proteome (1)	Mean # of genes (Std Dev)	4578 (248)	4895 (311)	4794 (199)	4643 (194)	4708 (247)	4675 (249)	4525 (240)	4683 (286)
Proteome woMGE (1)		4243 (154)	4419 (214)	4353 (108)	4291 (124)	4321 (131)	4321 (158)	4229 (144)	4309 (174)
MGE (1)		334 (144)	475 (151)	441 (135)	352 (123)	387 (148)	355 (158)	296 (144)	375 (158)
Sequence Type ST	Richness (NA*)	126 (5)	65 (11)	39 (4)	67 (1)	41 (1)	91 (5)	172 (10)	442 (38)
	α diversity(**)	6.61	5.29	3.66	5.45	4.40	6.29	6.83	7.53
H-type	Richness (NA*)	37 (1)	33	17	30 (5)	21 (3)	31 (2)	39 (1)	46 (15)
	α diversity(**)	4.87	4.32	2.93	4.16	3.22	4.59	4.74	4.73
O-group	Richness (NA*)	49 (101)	47 (163)	23 (24)	31 (73)	25 (46)	45 (45)	75 (105)	142 (563)
	α diversity(**)	5.20	5.00	3.43	4.65	4.36	4.88	5.20	5.91
O:H serotype	Richness (NA*)	78 (101)	66 (163)	30 (25)	46 (77)	30 (46)	65 (46)	108 (106)	311 (568)
	α diversity(**)	6.16	5.61	4.12	5.27	4.59	5.85	6.22	7.53
Average-genome (1)	# of gene families***	4504	4764	4685	4562	4602	4591	4454	4589
Pan-genome		30150	28576	19253	22284	20885	28391	34687	75890
α heaps law		0.44	0.54	0.59	0.64	0.57	0.42	0.49	0.46
Persistent-genome		2379	2474	2801	2572	2020 (4)	2599	2515	2486
Pan/Average		7	6	4	5	5	6	8	17
Persistent/Average %		53	52	60	56	44	57	56	54
MGE		11049	10382	6317	7668	6956	9802	12568	28651
MGE/Pan %		37	36	33	34	33	35	36	38
Phage-related (1)	Mean # of genes (Std Dev)	213 (90)	254 (95)	275 (102)	206 (79)	220 (102)	235 (111)	208 (99)	228 (99)
Plasmid-related (1)		141 (102	245 (120)	168 (75)	145 (96)	164 (103)	124 (98)	97 (90)	157 (113)
IS-related (1)		29 (14)	54 (19)	41 (11)	33 (17)	43 (20)	31 (19)	26 (13)	37 (19)
VFA (2)		105 (15)	108 (14)	118 (17)	116 (17)	113 (20)	112 (16)	103 (14)	109 (16)
VFB (2)		219 (29)	230 (26)	244 (33)	233 (30)	227 (36)	229 (26)	218 (25)	227 (29)
ARG Resfinder (2)		2.1 (2.1)	2.9 (2.0)	3.7 (3.2)	2.7 (2.8)	2.6 (2.5)	1.2 (0.8)	1.3 (1.2)	2.2 (1.2)
ARG Argannot (2)		5.1 (2.7)	5.8 (2.3)	6.2 (3.3)	5.3 (3.1)	5.3 (2.7)	3.8 (1.1)	4.1 (1.4)	5.0 (2.5)
int1+ (3)	% of genomes	7%	24%	40%	20%	20%	<1%	2%	14%
CONJ+ (2)		48%	66%	64%	57%	55%	52%	41%	54%
Rarefied-PanGenome (1,5) N=50	Mean # of gene families (Std Dev)	16141 (551)	15355 (502)	13837 (520)	14423 (483)	16099 (425)	17800 (557)	15497 (580)	
Rarefied-Persistent (1,5) N=50		2486 (71)	2488 (86)	2601 (61)	2628 (64)	2390 (60)	2486 (79)	2506 (91)	

* number of untypable genomes (NA)
** Shannon index
*** matrix of presence/absence of gene families : gene amplifications were not taken into account
**** mean of 112 core genes (the same as those of the 3 datasets : ECOR, RefSeq and Australian)
(1) standard ANOM test
(2) non-parametric ANOM test
(3) ANOM for proportions
(4) # of genomes <100; hence persistent genome = core genome in this case.
(5) rarefied datasets were computed from 1000 combinations of 50 distinct genomes.
