# Supplementary material for: Phylogenetic background and habitat drive the genetic diversification of Escherichia coli
Source: PLoS Genet. 2020 Jun 12;16(6):e1008866. doi: 10.1371/journal.pgen.1008866 (PMC7314097; doi:10.1371/journal.pgen.1008866)
Supplement: S6 Table — Same approach described in S3 Table. (PDF) [file pgen.1008866.s009.pdf]

1- STEPWISE REGRESSION REPORT

Y	SSE (1)	DFE (2)	RMSE (3)	RSquare (4)	Rsquare Adj (5)	Cp (6)	p (7)	AICc (8)	BIC (9)
MGE content	25850780.4	1269	142.7270	0.190	0.187	10.577	6	16276.75	16312.71

(1) SSE : Sum of squared errors for the current model

(2) DFE : Error degrees of freedom for the current model

(3) RMSE : Root mean square error (residual) for the current model

(4) RSquare : Proportion of the variation in the response that can be attributed to terms in the model rather than to random error.

(5) RSquare Adj: Adjusts R2 to make it more comparable over models with different numbers of parameters by using the degrees of freedom in its computation.
The adjusted R2 is useful in stepwise procedure because you are looking at many different models and want to adjust for the number of terms in the model.

(6) Cp : Mallow's Cp criterion for selecting a model.

(7) p : Number of parameters in the model, including the intercept.

(8) AICc : Corrected Akaike's Information Criterion

(9) BIC : Bayesian Information Criterion

2-STEP HISTORY REPORT

Y	Step		Parameter (12)	ACTION (10)	"Sig Prob" (11)	RSquare (4)	Cp (6)	p (7)	AICc (8)	BIC (9)
**MGE content**	**1**	Source	**Source{Water&BF&HI&MF&HF-HE&PM}**	**Entered**	**0**	**0.147**	**69.924**	**2**	**16334.38**	**16349.81**
**MGE content**	**2**	Phylogroup	**Phylogroup{B1&A&E-D&F&B2&G}**	**Entered**	**0**	**0.166**	**41.642**	**3**	**16307.27**	**16327.84**
**MGE content**	**3**	Source	**Source{Water-BF&HI&MF&HF}**	**Entered**	**0**	**0.179**	**23.314**	**4**	**16289.35**	**16315.06**
**MGE content**	**4**	Source	**Source{HE-PM}**	**Entered**	**0.003**	**0.185**	**16.396**	**5**	**16282.53**	**16313.37**
**MGE content**	**5**	Source	**Source{BF&HI&MF-HF}**	**Entered**	**0.0053**	**0.190**	**10.577**	**6**	**16276.75**	**16312.71**
MGE content	6	Phylogroup	Phylogroup{B1-A}	Entered	0.027	0.195	7.337	8	16273.53	16319.75
MGE content	7	Phylogroup	Phylogroup{B2-G}	Entered	0.2778	0.196	8.775	10	16275.02	16331.47
MGE content	8	Phylogroup	Phylogroup{D-F}	Entered	0.2716	0.197	9.567	11	16275.84	16337.40
MGE content	9	Source	Source{BF-HI&MF}	Entered	0.6634	0.197	11.377	12	16277.69	16344.36
MGE content	10	Source	Source{HI-MF}	Entered	0.5392	0.197	13.000	13	16279.35	16351.13
**MGE content**	**11**		**Best model**	**Specific**		**0.190**	**10.577**	**6**	**16276.75**	**16312.71**

(10) ACTION : Entered = Indicates whether a term is currently in the model.

(11) "Sig Prob" : The significance level associated with the Wald/Score ChiSq test statistic based on nDF degrees of freedom. The "Sig Prob" is used to determine the next term to be included in the model.

(12) Parameter : Water=freshwater; BF=Bird Faecal; HI=Human Intestinal, MF = Mammal Faecal; HF = Human Faecal ; HE = Human Extra-intestinal; PM = Poultry Meat

3-CURRENT ESTIMATES REPORT

Y	ACTION (10)		Parameter (12)	Estimate (13)	nDF (14)	SS (15)	"F Ratio" (16)	"Prob>F" (17)	RSquare (4)	%Explained_ Variance
MGE content			Intercept	395.206327	1	0	0	1		
**MGE content**	**Entered**	Phylogroup	**Phylogroup{B1&A&E-D&F&B2&G}**	**-21.028331**	**1**	**524366.694**	**25.741**	**4.49E-07**	**0.019**	**10.2**
MGE content		Phylogroup	Phylogroup{B1&A-E}	0	1	8643.56979	0.424	0.51500869		
MGE content		Phylogroup	Phylogroup{B1-A}	0	2	146965.821	3.622	0.02700251		
MGE content		Phylogroup	Phylogroup{D&F-B2&G}	0	1	10004.7586	0.491	0.48364091		
MGE content		Phylogroup	Phylogroup{D-F}	0	2	32196.297	0.79	0.45407397		
MGE content		Phylogroup	Phylogroup{B2-G}	0	2	51206.4187	1.257	0.28475944		
**MGE content**	**Entered**	Source	**Source{Water&BF&HI&MF&HF-HE&PM}**	**-59.923462**	**4**	**5353107.24**	**65.695**	**1.51E-50**	**0.147**	**77.4**
**MGE content**	**Entered**	Source	**Source{Water-BF&HI&MF&HF}**	**-30.671433**	**2**	**552870.98**	**13.57**	**1.47E-06**	**0.013**	**6.8**
**MGE content**	**Entered**	Source	**Source{BF&HI&MF-HF}**	**-22.50959**	**1**	**158694.835**	**7.79**	**0.00533165**	**0.005**	**2.6**
MGE content		Source	Source{BF-HI&MF}	0	1	11332.9911	0.556	0.45595975		
MGE content		Source	Source{HI-MF}	0	2	17104.9559	0.419	0.65749817		
**MGE content**	**Entered**	Source	**Source{HE-PM}**	**-24.267943**	**1**	**183640.521**	**9.015**	**0.0027304**	**0.006**	**3.0**

(13) Estimate :The current parameter estimate, which is zero if the effect is not currently in the model

(14) nDF : The number of degrees of freedom for a term. A term has more than one degree of freedom if its entry into a model also forces other terms into the model.

(15) SS : The reduction in the error (residual) sum of squares (SS) if the term is entered into the model or the increase in the error SS if the term is removed from the model.

(16) F ratio: The traditional test statistic to test that the term effect is zero. It is the square of a t-ratio.

(17) Prob>F : The significance level associated with the F statistic.
