# Supplementary material for: Phylogenetic background and habitat drive the genetic diversification of Escherichia coli
Source: PLoS Genet. 2020 Jun 12;16(6):e1008866. doi: 10.1371/journal.pgen.1008866 (PMC7314097; doi:10.1371/journal.pgen.1008866)
Supplement: Attachment — Submitted filename: Reviewer-MT-PlosG-1205.docx [file pgen.1008866.s032.docx]

**Answers to the Reviewers**

**Reviewer 1**

*The authors present a fantastic analysis of genetic diversity and pan genome dynamics in E. coli. I thoroughly enjoyed reading this paper and have no doubt it will make a major contribution to the E. coli community and the burgeoning scientific discourse on bacterial pan genomes.*

We thank Prof McNally for his appreciation of our work and pointing out the relevance of the topic.

## *I have a few comments the authors may wish to consider:*

## Comment n°1.1:

## *Line 86: In consequence beginning to sentence does not read well.*

**Answer to comment n°1.1:**

This was corrected.

Comment n°1.2 :

*Line 120: what are essentially core genes are given a new term persistent. Shouldnt they be termed Core to avoid confusion and align with common terminology.*

**Answer to comment n°1.2:**

The usual definition of core genome is the set of genes that are present in all (100%) of the genomes. Although recent works have broadened this definition to include less strict thresholds, we prefer not to do it for clarity.

In this study, the dataset is very large (with around 1300 genomes) and the genomes are essentially draft assemblies. The core genome, as defined above, is very small (~300 core-genes). This result reflects a biological reality, but also the quality of the sequencing, assembly and annotation of the genomes. The higher the number of genomes, the greater the impact of these different steps on the identification of core genes. If each genome has one core gene with an error, adding ca. 1300 means that the core genome falls to values close to zero. Hence, we had to be less stringent in allowing these genes to be absent in up to 1% of the genomes and called them persistent genes. This follows the literature, not only our previous papers, e.g. Gautreau, PlosCB, 2020, but also other authors like Acevedo-Rocha, TiG, 2013.

To make the phylogenetic trees, we used persistent genes, because it is possible to reconstruct a tree with a few genes missing in the multiple alignment of each gene family (which are replaced by gaps) and it was important to have a sufficiently large multiple alignment concatenated to obtain the best possible resolution of the trees. On the other hand, to calculate the nucleotide diversity, we used the core genes because the different programs available did not allow for missing data or gave questionable results.

Hence, in order to avoid any confusion, we have preferred, and still prefer, to use the two separate concepts of core and persistent genes (present in at least 99% of genomes), even if they correspond to “essentially core genes” as mentioned by the reviewer.

**Action**. We have made this point clearer in the text (first subsection of the Results), where the concept was not well clear and the distinction between core and persistent was vague.

Comment n°1.3:

*Line 157-198: Apologies as I see how much work this analysis has been. But this GRR finding is leaving me with a lot of questions. Is this differences in functional gene classes? Is it deviation in sequence of
similar genes as a result of drift? I feel i would like more info on the GRR diversification and description of the results.*

**Answer to comment n°1.3:**

The GRR variation is due to the variation in accessory genes (by definition singletons exist in one single genome and persistent genes are nearly ubiquitous).

Homologous gene families of the pan-genome were defined by clustering using a threshold of 80% sequence identity between pairs of homologs. Apart from this, the GRR does not account for the sequence similarity between genes of the same family, it just accounts for the relative frequency of genes of similar families across the pairs of genomes. For the GRR to be affected by sequence divergence, this should be so high that orthologs would become less than 80% identical (and thus fall in different families of the pan-genome decreasing the value of GRR). Yet, the observed average sequence identity is 98.3% across families of persistent genes (Fig. 1A). The average minimal identity value observed across the gene families was 95.5% (Fig. 1B), and there are very few genes families with sequence similarity between 80% and 95%. Hence, the probability that a gene evolves so fast that it becomes too divergent to be classed in the same family is very low. Stated in another way, a classical divergence process involving mutation accumulation, recombination, purifying selection and drift cannot explain the differences in terms of the GRR index. Of course, we cannot exclude the existence of a few extreme such cases of diversifying selection that lead a gene to diverge so fast that it would be less than 80% identical to its orthologs, but their number must be very small and would not affect the GRR values at the extent we observe (92% on average within STs). Hence, our results do not support the hypothesis that the gene repertoire variation is mainly due to the genetic drift of the ancestral sequences.

*Fig 1.* ***Sequence identity of the persistent genome.*** *Violin-plots of the average sequence identity (****A****), and the minimal sequence identity (****B****) observed in each of the 2,486 persistent gene families.*

To get a better understanding of the accessory genes affecting the GRR, we searched for functional EggNOG categories over-represented in accessory genes (Fig. 2).

*Fig 2.* ***Functional categories of the pan-genome****. Observed/expected (O/E) ratios of non-supervised orthologous groups (NOGs, shown as capitalized letters) in the three categories of gene families. The ratio (O/E) was reported for all comparisons with a color code ranging from blue (under-representation) to red (over-representation). The level of significance of each Fisher’s exact test was indicated (P>=0.05 : ns ; P<0.05 : * ; P<0.01 : ** ; P<0.001 :***). It was performed on each 2*2 contingency table. Only gene families with known functions were considered in this analysis.*

Action taken. Fig 1 was added to the supplementary material (see S2E Fig) and Fig 2 in the main text (see Fig 1C). Succinct explanations to these points were added to the results.

Comment n°1.4:

*Line 254: The suggestion of B2 not being basal to the tree. Isn’t this counter to the widely accepted pattern of evolution proposed by some of the authors of this paper, that B2 is the ancestral E. coli and A/B1 emerged from ancestral pathogens.*

**Answer to comment n°1.4:**

We are not completely certain of which previous statements this comment is about. But we agree there was some ambiguity in the text and in previous literature. The definition of basal is always a tricky one. Typical phylogenetic trees (like ours) are made of binary splits and therefore there are always two basal clades. One usually uses this expression to indicate one clade that is most divergent relative to a focal clade. In this sense, B2 has usually been regarded as basal relative to B1, D and A. Our tree shows clearly an initial split between B2/F/G on one side and the rest of the phylogroups on the other, and this could be interpreted as B2 being basal relative to others. However, since B2 does not stand alone in this initial partition of the tree, its description as basal may be misleading.

**Action.** We have discussed this topic more extensively in the discussion. The goal was to emphasize these results and how they fit with previous observations. The discussion also integrates this phylogenetic information with the data on gene repertoires that suggests that B2 change slower. This may give the impression that D/B1 arise from a B2 background. Actually, what this means is that the gene repertoires of B2 may be more similar to those of the LCA of *E. coli*. This seems consistent with the spirit of previous observations. We have not made statements about basal clades in this manuscript because of the comments stated above. We also have not compared these results with our previous results in Touchon, PlosG, 09, because there were no strains F and G at the time.

Comment n°1.5

*Line. 263: not probably more frequent. Absolutely more frequent*

**Answer to comment n°1.5:**

This was corrected.

Comment n°1.6

*Line 310-11: I am slightly confused at what level the prevalence of MGE is being described and analysed, and that no MGE is found in the core genome. Is this only true across the entire species data set? There are no MGEs which are core in a given lineage/phylogroup? Similarly in the following lines why would one expect MGE fixed across the species given the ecology of lineages is so varied? I am a bit confused by this section.*

**Answer to comment n°1.6:**

As MGEs were very often split in draft assemblies and it is well known that they are very diverse and rapidly evolving (especially in terms of gene content), it was not possible to reconstruct confidently the entire MGEs in such a large dataset. Hence, we have analyzed the distribution of the genes (or gene families) of these elements in all genomes (or in the pan-genomes). This is conservative, since different MGEs with a few very similar genes will lead to an artificial increase in the prevalence of these genes in the pan-genome, even if the MGEs were actually different. The result is a bias towards higher frequency of these genes. Nevertheless, as indicated in the main text, we found no gene families encoding for MGEs in the persistent genome of the full species. We also did not find MGEs gene families in the persistent genomes of the different phylogroups. However, we note that some **types** of MGEs are nearly ubiquitous (ISs, prophages, plasmids, as indicated in the text), they are just not homologous (different genomes have different prophages).

**Action**. We edited the text for clarity.

Comment n°1.7

*Line 417: potential number of possible sources is absolutely a function of phylogroup is it not?*

**Answer to comment n°1.7:**

All possible sources are found in all the different phylogroups (see Fig 6A), even if some appear to be more associated with peculiar phylogroups (see Fig 6B).

**Action**. We edited the text for clarity.

Comment n°1.8

*Line 636: I can only assume the 454 genomes represents the fact some genomes were sequenced as legacy projects some time ago. But to fend off criticism I would provide a very clear and honest assessmentt of how why and when these were sequenced.*

**Answer to comment n°1.8:**

70 genomes were sequenced at Broad institute using the Roche 454 GS FLX system. This sequencing was an effort to generate an ‘improved’ ECOR collection, by including strains that were largely commensal and isolated from both human and non-human sources, and encompassed the diversity of *E. coli*.   A sub-goal was to compare and contrast the genomes of ST10 and ST95 which we knew to be two successful human associated lineages. The strains were sequenced at least 10 years ago and 454 was the most appropriate technology at the time.

**Action**. We edited the text for clarity, added details and included references to previous articles where methods were described.

**Reviewer 2**

*E. coli can be found in all endothermic animals and is consequently associated with their environment (water, soil, plants). Currently, there is a strong bias towards clinical samples in E. coli studies. Here, authors use an impressive sampling of >5,000 isolates to sequence and characterize a subset of representative >1,200 E. coli from mostly non-clinical, isolates originating from humans, poultry, wild animals and water sampled from the Australian continent (no plants). Using comparisons of pangenomic diversity and gene repertoires, authors show that (1) there are large variations in gene content in E. coli lineages, even within STs, (2) gene flow explains the association of certain lineages with specific ecological niches and habitats, something that is not new, but had never been looked at in such way, (3) smaller genomes are associated with a higher (and not lower) turnover of genetic information, and (4) an interesting proportion of small genomes were freshwater isolates, which is very interesting and provides new evidence suggesting that E. coli can be "naturalized" and adapt/replicate in non-host environments.

The results from this study are not ground-breakingly novel. It's been more than a decade that we (including many studies from authors of this manuscript) find evidence for phylogroup-specific ecologies and physiologies in E. coli but it is the first study of this scale, which makes it seminal and authoritative.*

We thank the Reviewer for her/his appreciation of our work.

Overall, I enjoyed this manuscript a lot, and will be happy to recommend it for publication in Plos Genetics after a few clarifications are made:


Comment n°2.1

Methods: *I think I need some clarification on the phylogenetics section, because I am a bit confused as to why the authors chose to do things the way they did. I would understand that aligning with E-INS-i in MAFFT is not recommended for >200 sequences, but is it why authors used this odd strategy of back-translating concatenated protein-to-protein alignments instead of performing the alignment on gene-by-gene concatenates?I am also missing details as to how the back-translation was performed: which codon usage did you pick? How is that affecting recombination/HGT/diversity analyses you are doing afterwards? Or did you come back to the original nucleotide sequence? At the very least, a clarification of the rationale here would be more than welcome.*

**Answer to comment n°2.1:**

We used mafft v.7.222 with FFT-NS-2 option because it’s a **fast**-progressive method recommended for **large datasets** (Nakamura, Bioinformatics, 2018). One should note that since members of the same gene families are on average 98% identical, the alignments are very accurate even with this method.

All the phylogenetic trees were performed from **gene-by-gene concatenates**. We would like to thank the reviewer for pointing out this misunderstanding related to the same error indicated line 766 and 786.

-(line 766): “*The phylogenetic species tree of Australian E. coli was reconstructed from the concatenated alignments of the 2,486* ***persistent genes*** *of the 1,294 Australian E. coli strains”.*

*-(line 786): “In this case, the persistent-genome is composed of 1,589* ***gene families****, and the resulting alignment of 1,469,523 bp”.*

We made the alignments from the protein sequences because these alignments are known to be more reliable (larger alphabet and smaller sequence divergence between proteins than between the corresponding DNA sequences). We then back-translated them using the **original DNA sequences**, as is standard usage. Within a species, this does not affect very much the final alignments, but we wanted to use the same approach for the analysis of the Escherichia clade that are not *E. coli* and are used as outgroups. These genomes are more distant and might have been less well aligned when using DNA sequences. We want to comfort the reviewer and confirm that this step of back-translation has **no impact** on the HGT/diversity analyses.

**Action**. The methods section was changed to accommodate these explanations.

Comment n°2.2

*Similarly, another amber flag (to not say red) for me was to read that authors manually added gaps "-" in the alignment. Please explain a bit better.*

**Answer to comment n°2.2:**

As mentioned above (comment 1.2), due to the size of the dataset and the draft status of genome assemblies (not assembled into complete replicons), the core genome (set of genes present in 100% of genomes) is very small (around 300 core genes). This number is small to resolve a tree containing 1294 tips. It is then best to use larger alignments, because adding genes to the alignment augments the number of informative sites available for estimating divergence events deep in the tree and improves their time estimates. Therefore, we made the tree with the persistent genes (which are present in at least 99% genomes). Thus, in most families of persistent genes (88%), a gene is missing in 1 up to 12 genomes, but it’s important to note that missing genes account for only **0.3%** of the genes in the alignment.

So, we made the alignment (A) with all the available genes of the (F_A_) family, then we **automatically** added a series of gaps (of the length of the alignment F_A_) for all the genomes in which the gene was missing. This is the only way to be able to then concatenate the different gene families’ multiple alignments (F_n_) which must contain the same number of genomes. In the resulting multiple alignment concatenated, the average number of gaps per genome is around 0.3%, and the maximal observed is around 3% of the aligned sequence. Hence, the amount of missing data is very small, widely scattered throughout the different gene families and genomes, and thus should have little or no impact on the reconstruction of the tree. This is a standard procedure, and it was shown to be reliable by several papers. For example, Filipski et al (Mol Biol Evol, 2014) showed that this approach adds significant information to the phylogenetic reconstruction when sparseness (fraction of missing data in the gene-by-species matrix) is as high as 60% (i.e. around 2000 times higher than in our study).

**Action.** We have made this point clearer and added a supplementary figure (S3 Fig) which illustrates the different steps of the concatenation of multiple alignments of persistent gene families.

Comment n°2.3

*I couldn't access the sequences on ENA accession PRJEB34791 nor on the SRA using the individual accessions from the S1 dataset. Please make sure to make things available when the paper is published, or for eventual future reviews.*

**Answer to comment n°2.3:**

The assemblies were deposited into the European Nucleotide Archive (PRJEB34791). The accession number of each genome is reported in S1 Dataset. We have now requested to make all the sequences publicly available.

Comment n°2.4

*I would have liked to see a bit more details on the sources of isolations presented in the S1 Dataset. Which birds and non-human mammals were actually sampled? Were they captive or wild?*

**Answer to comment n°2.4:**

We have now added these details to the S1 Dataset.

Comment n°2.5

*It would be very good to provide with more information on the actual animal source for each isolate, rather than just being vague and mention "bird faecal" for example. I suspect there is a collection of various Australian animals there, but at the moment we cannot tell.*

**Answer to comment n°2.5:**

The reviewer is right, this dataset contains a very large variety of Australian animals. This information is now shown in the S1 Dataset.

Comment n°2.6

*Results: I would have liked to see more genetics in this analysis: you have characterised the pangenome of a rather unique dataset in one of the most iconic bacterial species ever studied, and yet, I am left hungry for gene names, and knowing which functions you find to be potentially involved in what. For instance, what functions would be phylogroup-specific in healthy and environmental E. coli? Are they predicted metabolic functions like we expect?*

**Answer to comment n°2.6:**

Most phylogroups lack specific gene families, i.e., gene families present in all strains and absent from the rest of the species. As requested by the reviewers, we inferred the gene families associated to a phylogroup using a GWAS approach and found many gene families which are now described in S2 Dataset.

**Action.** We have added the GWAS analyses to the main text, as well as the figure (Fig 7). A complete list of gene families (including functional annotations and reference gene sequences) positively and negatively associated to each phylogroup is now described in S2 Dataset.

Comment n°2.7

*Discussion: - I find the generalism of phylogroup B1 fascinating, so I enjoyed the freshwater angle at the end of this study. Instead of an open sentence at the end of the manuscript, would authors care to hazard a more exhaustive interpretation on the evolution of phylogroup B1? A majority of B1 isolates are also found in ruminants and on plants, which could maybe have an obvious link with freshwater composition too. The deadliest outbreak strain of E. coli (O104:H4) was also a B1 isolate. Is B1 only the most dramatic example (i.e. for which we can find a possible explanation) of the impact of ecology on phylogroup evolution?*

**Answer to comment n°2.7:**

To answer to the reviewer, we made a GWAS analysis. This revealed a number of interesting metabolic traits associated with B1 strains that go in the direction proposed, e.g. associated with compounds found in plants. This was added to the manuscript in the results section and commented in the discussion.

Comment n°2.8

*- Linked to my comment above for the Results section, I would have liked to see a bit more on the possible genetic functions involved in the "naturalization" in the freshwater niche (to paraphrase Mike Sadowsky). Are these functions also found in other bacteria that are found in natural environments? Can this study contribute to a better idea of what is actually E. coli associated with in freshwater (free-living vs. surviving in protozoa vs. in sediments)? If I recall well, this is still quite an unclear point.*

 **Answer to comment n°2.8**

As mentioned above, we made a GWAS to determine genetic determinants associated to each source. This revealed genes and pathways associated with specific sources.

**Action.** We have added the GWAS, succinctly mention key functions in the text, and added two figures (Fig 8 and S20 Fig) to illustrate these results. A complete list of gene families positively and negatively associated to each isolation source is now described in S2 Dataset.
