# Supplementary material for: Phylogenetic background and habitat drive the genetic diversification of Escherichia coli
Source: PLoS Genet. 2020 Jun 12;16(6):e1008866. doi: 10.1371/journal.pgen.1008866 (PMC7314097; doi:10.1371/journal.pgen.1008866)
Supplement: Peer review history

# Peer review: Decision Letter 0

**DOI:** 10.1371/journal.pgen.1008866.r001

**Authors:** Kirsten Bomblies, Xavier Didelot

15 Apr 2020

* Please note while forming your response, if your article is accepted, you may have the opportunity to make the peer review history publicly available. The record will include editor decision letters (with reviews) and your responses to reviewer comments. If eligible, we will contact you to opt in or out. *

Dear Dr Touchon,

Thank you very much for submitting your Research Article entitled 'Phylogenetic background and habitat drive the genetic  diversification of Escherichia coli' to PLOS Genetics. Your manuscript was fully evaluated at the editorial level and by independent peer reviewers. The reviewers appreciated the attention to an important topic but identified some aspects of the manuscript that should be improved.

We therefore ask you to modify the manuscript according to the review recommendations before we can consider your manuscript for acceptance. Your revisions should address the specific points made by each reviewer.

In addition we ask that you:

1) Provide a detailed list of your responses to the review comments and a description of the changes you have made in the manuscript.

2) Upload a Striking Image with a corresponding caption to accompany your manuscript if one is available (either a new image or an existing one from within your manuscript). If this image is judged to be suitable, it may be featured on our website. Images should ideally be high resolution, eye-catching, single panel square images. For examples, please browse our archive. If your image is from someone other than yourself, please ensure that the artist has read and agreed to the terms and conditions of the Creative Commons Attribution License. Note: we cannot publish copyrighted images.

We hope to receive your revised manuscript within the next 30 days. If you anticipate any delay in its return, we would ask you to let us know the expected resubmission date by email to plosgenetics@plos.org.

If present, accompanying reviewer attachments should be included with this email; please notify the journal office if any appear to be missing. They will also be available for download from the link below. You can use this link to log into the system when you are ready to submit a revised version, having first consulted our Submission Checklist.

While revising your submission, please upload your figure files to the Preflight Analysis and Conversion Engine (PACE) digital diagnostic tool. PACE helps ensure that figures meet PLOS requirements. To use PACE, you must first register as a user. Then, login and navigate to the UPLOAD tab, where you will find detailed instructions on how to use the tool. If you encounter any issues or have any questions when using PACE, please email us at figures@plos.org.

Please be aware that our data availability policy requires that all numerical data underlying graphs or summary statistics are included with the submission, and you will need to provide this upon resubmission if not already present. In addition, we do not permit the inclusion of phrases such as "data not shown" or "unpublished results" in manuscripts. All points should be backed up by data provided with the submission.

PLOS has incorporated Similarity Check, powered by iThenticate, into its journal-wide submission system in order to screen submitted content for originality before publication. Each PLOS journal undertakes screening on a proportion of submitted articles. You will be contacted if needed following the screening process.

To resubmit, you will need to go to the link below and 'Revise Submission' in the 'Submissions Needing Revision' folder.

[LINK]

Please let us know if you have any questions while making these revisions.

Yours sincerely,

Xavier Didelot

Associate Editor

PLOS Genetics

Kirsten Bomblies

Section Editor: Evolution

PLOS Genetics

Reviewer's Responses to Questions

**Comments to the Authors:**

**Please note here if the review is uploaded as an attachment.**

Reviewer #1: The authors present a fantastic analysis of genetic diversity and pan genome dynamics ics in E. coli. I thoroughly enjoyed reading this paper and have no doubt it will make a major contribution to the E. coli community and the burgeoning scientific discourse on bacterial pan genomes. I have a few comments the authors may wish to consider:

Line 86: In consequence beginning to sentence does not read well

Line 120: what are essentially core genes are given a new term persistent. Shouldnt they be termed Core to avoid confusion and align with common terminology

Line 157-198: Apologies as I see how much work this analysis has been. But this GRR finding is leaving me with alot of questions. Is this differences in functional gene classes? Is it deviation in sequence of

similar genes as a result of drift? I feel i would like more info on the GRR diversification and description of the results.

Line 254: The suggestion of B2 not being basal to the tree. Isnt this counter to the widely accepted pattern of evolution proposed by some of the authors of this paper, that B2 is the ancestral E. coli and A/B1 emerged from ancestral pathogens

Line. 263: not probably more frequent. Absolutely more frequent

Line 310-11: I am slightly confused at what level the prevalence of MGE is being described and analysed, and that no MGE is found in the core genome. Is this only true across the entire species data set? There are no MGEs which are core in a given lineage/phylogroup? Similarly in the following lines why would one expect MGE fixed across the species given the ecology of lineages is so varied? I am a bit confused by this section.

Line 417: potential number of possible sources is absolutely a function of phylogroup is it not?

Line 636: I can only assume the 454 genomes represents the fact some genomes were sequenced as legacy projects some time ago. But to fend off criticism I would provide a very clear and honest assessmentt of how why and when these were sequenced.

Reviewer #2: Dear Professor Didelot,

First of all I apologise for the delay in my review. Disruptions and subsequent preparations linked to the COVID-19 pandemic have got in the way of providing you with a quick review. I have now read with attention the manuscript submitted to you by Marie Touchon et al. entitled "Phylogenetic background and habitat drive the genetic diversification of Escherichia coli"

E. coli can be found in all endothermic animals and is consequently associated with their environment (water, soil, plants). Currently, there is a strong bias towards clinical samples in E. coli studies. Here, authors use an impressive sampling of >5,000 isolates to sequence and characterize a subset of representative >1,200 E. coli from mostly non-clinical, isolates originating from humans, poultry, wild animals and water sampled from the Australian continent (no plants). Using comparisons of pangenomic diversity and gene repertoires, authors show that (1) there are large variations in gene content in E. coli lineages, even within STs, (2) gene flow explains the association of certain lineages with specific ecological niches and habitats, something that is not new, but had never been looked at in such way, (3) smaller genomes are associated with a higher (and not lower) turnover of genetic information, and (4) an interesting proportion of small genomes were freshwater isolates, which is very interesting and provides new evidence suggesting that E. coli can be "naturalized" and adapt/replicate in non-host environments.

The results from this study are not ground-breakingly novel. It's been more than a decade that we (including many studies from authors of this manuscript) find evidence for phylogroup-specific ecologies and physiologies in E. coli but it is the first study of this scale, which makes it seminal and authoritative.

Overall, I enjoyed this manuscript a lot, and will be happy to recommend it for publication in Plos Genetics after a few clarifications are made:

Methods:

- I think I need some clarification on the phylogenetics section, because I am a bit confused as to why the authors chose to do things the way they did. I would understand that aligning with E-INS-i in MAFFT is not recommended for >200 sequences, but is it why authors used this odd strategy of back-translating concatenated protein-to-protein alignments instead of performing the alignment on gene-by-gene concatenates? I am also missing details as to how the back-translation was performed: which codon usage did you pick? How is that affecting recombination/HGT/diversity analyses you are doing afterwards? Or did you come back to the original nucleotide sequence? At the very least, a clarification of the rationale here would be more than welcome. Similarly, another amber flag (to not say red) for me was to read that authors manually added gaps "-" in the alignment. Please explain a bit better.

- I couldn't access the sequences on ENA accession PRJEB34791 nor on the SRA using the individual accessions from the S1 dataset. Please make sure to make things available when the paper is published, or for eventual future reviews.

- I would have liked to see a bit more details on the sources of isolations presented in the S1 Dataset. Which birds and non-human mammals were actually sampled? Were they captive or wild?

- It would be very good to provide with more information on the actual animal source for each isolate, rather than just being vague and mention "bird faecal" for example. I suspect there is a collection of various Australian animals there, but at the moment we cannot tell.

Results:

- I would have liked to see more genetics in this analysis: you have characterised the pangenome of a rather unique dataset in one of the most iconic bacterial species ever studied, and yet, I am left hungry for gene names, and knowing which functions you find to be potentially involved in what. For instance, what functions would be phylogroup-specific in healthy and environmental E. coli? Are they predicted metabolic functions like we expect?

Discussion:

- I find the generalism of phylogroup B1 fascinating, so I enjoyed the freshwater angle at the end of this study. Instead of an open sentence at the end of the manuscript, would authors care to hazard a more exhaustive interpretation on the evolution of phylogroup B1? A majority of B1 isolates are also found in ruminants and on plants, which could maybe have an obvious link with freshwater composition too. The deadliest outbreak strain of E. coli (O104:H4) was also a B1 isolate. Is B1 only the most dramatic example (i.e. for which we can find a possible explanation) of the impact of ecology on phylogroup evolution?

- Linked to my comment above for the Results section, I would have liked to see a bit more on the possible genetic functions involved in the "naturalization" in the freshwater niche (to paraphrase Mike Sadowsky). Are these functions also found in other bacteria that are found in natural environments? Can this study contribute to a better idea of what is actually E. coli associated with in freshwater (free-living vs. surviving in protozoa vs. in sediments)? If I recall well, this is still quite an unclear point.

**********

**Have all data underlying the figures and results presented in the manuscript been provided?**

Large-scale datasets should be made available via a public repository as described in the *PLOS Genetics* data availability policy, and numerical data that underlies graphs or summary statistics should be provided in spreadsheet form as supporting information.

Reviewer #1: Yes

Reviewer #2: No: I couldn't access the sequences on ENA accession PRJEB34791 nor on the SRA using the individual accessions from the S1 dataset.

**********

PLOS authors have the option to publish the peer review history of their article (what does this mean?). If published, this will include your full peer review and any attached files.

If you choose “no”, your identity will remain anonymous but your review may still be made public.

**Do you want your identity to be public for this peer review?** For information about this choice, including consent withdrawal, please see our Privacy Policy.

Reviewer #1: Yes: Alan McNally

Reviewer #2: No

# Author comment: Author response to Decision Letter 0

**DOI:** 10.1371/journal.pgen.1008866.r002

15 May 2020

Attachment Submitted filename: Reviewer-MT-PlosG-1205.docx Click here for additional data file.

# Editor report: Decision Letter 1

**DOI:** 10.1371/journal.pgen.1008866.r003

**Authors:** Kirsten Bomblies, Xavier Didelot

18 May 2020

Dear Dr Touchon,

We are pleased to inform you that your manuscript entitled "Phylogenetic background and habitat drive the genetic  diversification of Escherichia coli" has been editorially accepted for publication in PLOS Genetics. Congratulations!

Before your submission can be formally accepted and sent to production you will need to complete our formatting changes, which you will receive in a follow up email. Please be aware that it may take several days for you to receive this email; during this time no action is required by you. Please note: the accept date on your published article will reflect the date of this provisional accept, but your manuscript will not be scheduled for publication until the required changes have been made.

Once your paper is formally accepted, an uncorrected proof of your manuscript will be published online ahead of the final version, unless you’ve already opted out via the online submission form. If, for any reason, you do not want an earlier version of your manuscript published online or are unsure if you have already indicated as such, please let the journal staff know immediately at plosgenetics@plos.org.

In the meantime, please log into Editorial Manager at https://www.editorialmanager.com/pgenetics/, click the "Update My Information" link at the top of the page, and update your user information to ensure an efficient production and billing process. Note that PLOS requires an ORCID iD for all corresponding authors. Therefore, please ensure that you have an ORCID iD and that it is validated in Editorial Manager. To do this, go to ‘Update my Information’ (in the upper left-hand corner of the main menu), and click on the Fetch/Validate link next to the ORCID field.  This will take you to the ORCID site and allow you to create a new iD or authenticate a pre-existing iD in Editorial Manager.

If you have a press-related query, or would like to know about one way to make your underlying data available (as you will be aware, this is required for publication), please see the end of this email. If your institution or institutions have a press office, please notify them about your upcoming article at this point, to enable them to help maximise its impact. Inform journal staff as soon as possible if you are preparing a press release for your article and need a publication date.

Thank you again for supporting open-access publishing; we are looking forward to publishing your work in PLOS Genetics!

Yours sincerely,

Xavier Didelot

Associate Editor

PLOS Genetics

Kirsten Bomblies

Section Editor: Evolution

PLOS Genetics

www.plosgenetics.org

Twitter: @PLOSGenetics

----------------------------------------------------

Comments from the reviewers (if applicable):

----------------------------------------------------

**Data Deposition**

If you have submitted a Research Article or Front Matter that has associated data that are not suitable for deposition in a subject-specific public repository (such as GenBank or ArrayExpress), one way to make that data available is to deposit it in the Dryad Digital Repository. As you may recall, we ask all authors to agree to make data available; this is one way to achieve that. A full list of recommended repositories can be found on our website.

The following link will take you to the Dryad record for your article, so you won't have to re‐enter its bibliographic information, and can upload your files directly: 

http://datadryad.org/submit?journalID=pgenetics&manu=PGENETICS-D-20-00247R1

More information about depositing data in Dryad is available at http://www.datadryad.org/depositing. If you experience any difficulties in submitting your data, please contact help@datadryad.org for support.

Additionally, please be aware that our data availability policy requires that all numerical data underlying display items are included with the submission, and you will need to provide this before we can formally accept your manuscript, if not already present.

----------------------------------------------------

**Press Queries**

If you or your institution will be preparing press materials for this manuscript, or if you need to know your paper's publication date for media purposes, please inform the journal staff as soon as possible so that your submission can be scheduled accordingly. Your manuscript will remain under a strict press embargo until the publication date and time. This means an early version of your manuscript will not be published ahead of your final version. PLOS Genetics may also choose to issue a press release for your article. If there's anything the journal should know or you'd like more information, please get in touch via plosgenetics@plos.org.

# Editor report: Acceptance letter

**DOI:** 10.1371/journal.pgen.1008866.r004

**Authors:** Kirsten Bomblies, Xavier Didelot

4 Jun 2020

PGENETICS-D-20-00247R1 

Phylogenetic background and habitat drive the genetic diversification of Escherichia coli 

Dear Dr Touchon, 

We are pleased to inform you that your manuscript entitled "Phylogenetic background and habitat drive the genetic diversification of Escherichia coli" has been formally accepted for publication in PLOS Genetics! Your manuscript is now with our production department and you will be notified of the publication date in due course.

The corresponding author will soon be receiving a typeset proof for review, to ensure errors have not been introduced during production. Please review the PDF proof of your manuscript carefully, as this is the last chance to correct any errors. Please note that major changes, or those which affect the scientific understanding of the work, will likely cause delays to the publication date of your manuscript. 

Soon after your final files are uploaded, unless you have opted out or your manuscript is a front-matter piece, the early version of your manuscript will be published online. The date of the early version will be your article's publication date. The final article will be published to the same URL, and all versions of the paper will be accessible to readers.

Thank you again for supporting PLOS Genetics and open-access publishing. We are looking forward to publishing your work! 

With kind regards,

Matt Lyles

PLOS Genetics

On behalf of:

The PLOS Genetics Team

Carlyle House, Carlyle Road, Cambridge CB4 3DN | United Kingdom

plosgenetics@plos.org | +44 (0) 1223-442823

plosgenetics.org | Twitter: @PLOSGenetics
